# A Deep Auto-Optimized Collaborative Learning (DACL) model for disease prognosis using AI-IoMT systems

**DOI:** 10.1038/s41598-024-59846-2

**Published:** 2024-05-04

**Authors:** Malarvizhi Nandagopal, Koteeswaran Seerangan, Tamilmani Govindaraju, Neeba Eralil Abi, Balamurugan Balusamy, Shitharth Selvarajan

**Affiliations:** 1 Department of CSE, School of Computing, Vel Tech Rangarajan Dr. Sagunthala R&D Institute of Science and Technology, Chennai, Tamil Nadu 600062 India; 2 Department of CSE (AI&ML), S.A. Engineering College (Autonomous), Chennai, Tamil Nadu 600077 India; 3 Department of Computational Intelligence, SRM Institute of Science and Technology, Kattankulathur, Chennai, Tamil Nadu 603203 India; 4 Department of Information Technology, Rajagiri School of Engineering and Technology, Kochi, Kerala 682039 India; 5 Shiv Nadar (Institution of Eminence Deemed to be University), Greater Noida, Uttar Pradesh 201314 India; 6 Department of Computer Science, Kebri Dehar University, 250, Kebri Dehar, Ethiopia; 7 School of Built Environment, Engineering and Computing, Leeds Beckett University, LS1 3HE Leeds, UK

**Keywords:** Deep Auto-Optimized Collaborative Learning (DACL) model, Internet of Medical Things (IoMT), Disease diagnosis, Artificial Intelligence (AI), Data imputation, Optimization, Classification.1, Health care, Energy science and technology

## Abstract

In modern healthcare, integrating Artificial Intelligence (AI) and Internet of Medical Things (IoMT) is highly beneficial and has made it possible to effectively control disease using networks of interconnected sensors worn by individuals. The purpose of this work is to develop an AI-IoMT framework for identifying several of chronic diseases form the patients’ medical record. For that, the Deep Auto-Optimized Collaborative Learning (DACL) Model, a brand-new AI-IoMT framework, has been developed for rapid diagnosis of chronic diseases like heart disease, diabetes, and stroke. Then, a Deep Auto-Encoder Model (DAEM) is used in the proposed framework to formulate the imputed and preprocessed data by determining the fields of characteristics or information that are lacking. To speed up classification training and testing, the Golden Flower Search (GFS) approach is then utilized to choose the best features from the imputed data. In addition, the cutting-edge Collaborative Bias Integrated GAN (ColBGaN) model has been created for precisely recognizing and classifying the types of chronic diseases from the medical records of patients. The loss function is optimally estimated during classification using the Water Drop Optimization (WDO) technique, reducing the classifier’s error rate. Using some of the well-known benchmarking datasets and performance measures, the proposed DACL’s effectiveness and efficiency in identifying diseases is evaluated and compared.

## Introduction

The Internet of Medical Things (IoMT) intends to deliver innovative and affordable solutions to the medical field, especially by enabling safer observation of reliant patients or individuals who suffer from chronic illnesses^1,2^. The IoMT makes use of connected medical devices and portable technologies that are currently available on the end user market at reasonable rates. The incompatible nature of linked devices, the requirement to connect numerous systems, and data security provide major barriers to the application of IoMT solutions^3,4^. Developers of these applications ought to get granted tools that protect them against the degree of complexity brought on by these problems in order to fully utilize the possibilities of IoMT. The elderly population is increasing at an unheard-of rate. The United Nations projects that by 2030, people 65 and older will make up 15.7% of the entire population^5^. The significant rise in the number of elderly and feeble people has made it necessary to offer ongoing healthcare and remote health monitoring to ensure that these aging inhabitants may live engaged and autonomous lives without the assistance of personal aides. Because of its broad spectrum of capabilities and potential application areas, the Internet of Things (IoT)^6^ is a technology of opportunity which has attracted a lot of interest in the modern day. Through the integration of internet-connected gadgets with health care devices, the idea of the IoMT arose as a platform to offer cutting-edge remote medical treatment and telemedicine services^7,8^. The IoMT model ensures the followings:

- Precise remote health surveillance in real time with decreased mistakes;
- Lowered healthcare costs;
- Enhanced patient comfort and fulfillment;
- Time-efficiency by means of the abolition of painful waits and unnecessary medical appointments, especially in remote locations;
- More proficient diagnosis, early detection, and management;
- Optimized therapy results through enhanced administration of medications.

In affluent nations, the health sector is undergoing significant change as a result of the twentieth century's sudden increase in lifespans. The healthcare systems of these nations are also coming under increasing strain from chronic diseases. In fact, over the course of the twentieth century, the average life expectancy in wealthy nations increased by around 30 years^9^. As a consequence of this, the number of senior citizens has significantly risen. Furthermore, the rise in chronic diseases has put strain on healthcare systems globally as a result of an acute lack of funding. As the population ages and chronic diseases develop, healthcare systems must not only deal with a growing number of patients but also a wide range of illnesses and treatments. In-home telemedicine technology are being proven to be effective alternatives to minimize overtaxing medical facilities and to lower medical costs. Telemedicine systems, on the other hand, are incredibly diverse and typically created to tackle a specific medical objective, such as remote heart monitoring, rehabilitation after a stroke, etc.^10,11^. This feature of telemedicine networks allows them to be effective at cutting expenses and overburdening the medical system, although it constitutes a disadvantage as an increasing amount of patients and the range of ailments rise^12^. IoMT can address the need for more generality and manageability. Smart gadgets, and online resources are used by medical systems to acquire medical data directly and integrate people, services, and enterprises. A small number of people who favor patient monitoring include medical professionals, workers, businesses, and research institutions. This dynamic environment includes the detection and monitoring of diseases, decision-making processes, medical management, and medical expertise^13,14^.

In fact, the IoMT blends traditional IoT's dynamic, flexible, and scalability features with the dependability and safety of conventional medical devices^15–17^. By managing multiple gadgets deployed for numerous patients and being sufficiently generic to handle a variety of conditions calling for numerous monitoring and controlling demands, it has the potential to overcome the problem of aging and chronic diseases^18^. In addition, IoMT offers a solution for other problems including patient transportation (i.e., constant monitoring of patients in their daily activities as contrasted with telemedicine systems, which are primarily concerned with home-care). Considering the complexity of these problems, modern technological solutions for wealthy countries' intricate healthcare systems are revolutionizing the way healthcare is provided. The establishment of the IoMT and solutions to meet the demands of both the elderly as well as individuals with chronic diseases are being made possible by the expansion of personal computing devices and advances in their computational capacity. The IoMT^19^ involves the linking of many different personalized health equipment with healthcare organizations like medical facilities, academic institutions, and commercial entities. The necessities from the lower parts of the IoMT must be transferred to higher elements of the IoMT as the IoMT is characterized as the connectivity of medical-grade equipment with wider healthcare facilities. The interlinked nature of medical devices also introduces new requirements, such as analyzing and safeguarding gathered data, access to information policies, or management of data lifecycle standards^20–22^. Similar to the conventional IoT, IoMT devices come in a wide range of computing power, protocol specifications, along with application domains. Systems used for the IoMT have to be able to efficiently manage the vast number of sensors that make up the IoMT. As a result, the IoMT has similar requirements to the standard IoT, particularly when it comes to managing a large number of machines, ensuring consistent connection, or supporting heterogeneous and compatible devices.

The IoMT, AI, and big data are connected fields in customized medical field^23^ that are having a large influence on the development and construction of an improved system. In healthcare, integrating AI and IoMT is useful and has made it possible to effectively control disease using networks of interconnected sensors worn by individuals. IoT is a dynamic field of study in the area of disease management. AI-IoMT is a cutting-edge method of integrating medical devices and their uses to communicate with people as well as information innovation systems. It is necessary to investigate the potential effects of using the AI-IoMT technique to treat all patient groups equally, without showing any preference to the wealthy or those who are impoverished, in order to combat progressive diseases. Knowledge sharing, report authentication, study, monitoring of patients, information concerns, cleaning healthcare attention, and other IoT services are some of the different cloud-based IoT services.

IoT and AI-driven smart health care face several fundamental challenges, including connectivity of sensors, interaction among devices, safety and confidentiality of data, device management, data management obstacles, as well as successful utilization of AI. The majority of IoMT devices might be used for finding and diagnosing illnesses in various healthcare settings, and the data gathered from heterogeneous sensors comprises a number of issues, including failures in the hardware, inadequate battery life, and difficulties with communication. Some fundamental issues have become prevalent and unavoidable. Specifically, there are occasionally unexplained errors made when using common healthcare sensors like smartphones and wearable. In addition, there are standard complications like longevity of batteries, discriminating between specific physical features and changes in the environment. The aforementioned issues demonstrate that even with the usage of many IoMT devices and multisensory signals, there are still a number of challenges in the field of smart health care. To promote the widespread adoption of such smart healthcare, a simple and easy integration solution ought to be developed. In each smart system, there is a rise in the quantity of linked sensors, devices, and IoTs. The ability to generate significant amounts of data as well as perception is a prerequisite for a massive amounts healthcare system to continue functioning. Millions of interconnected sensors and IoT devices in the medical field offer enormous amounts of data for processing. The data architecture along with the knowledge description format adopted by the IoT entities should be consistent. The current review of the literature indicates that certain elements of conventional IoMT-enabled healthcare applications pose significant challenges: prolongation of the prognosis period for the disease, expensive cost, limited scalability and dependability, decreased effectiveness, reduced accuracy in the classification and identification of diseases.

Involving infectious disease diagnosis and monitoring has the ability to change how medical services are structured while simultaneously giving more patients access to better care and increased satisfaction^24,25^. As a kind of early warning, Internet of Things devices like the GPS have the potential to be a vital tool in halting the spread of such diseases. Temperature detectors and other indicators can be used to identify people who are ill. The following lists this research work's primary contributions:

- An innovative AI-IoMT architecture called the Deep Auto-Optimized Collaborative Learning (DACL) Model is intended to accurately identify chronic conditions like diabetes, stroke, and heart disease.
- In the proposed framework, the imputed and preprocessed data are formulated by using a Deep Auto-Encoder Model (DAEM) to find the fields of missing characteristics or data.
- The Golden Flower Search (GFS) technique is then used to select the best features from the imputed data, accelerating training and testing for classification.
- Furthermore, a novel model named Collaborative Bias Integrated GAN (ColBGaN) is developed to accurately identify and categorize the various types of chronic illnesses according to patient medical data.
- The Water Drop Optimization (WDO) technique is used to optimally approximate the loss function during classification, hence reducing the classifier's error rate.

The proposed framework illustrates the three tiers of the proposed AI-IoMT architecture. First, sensors are used to collect the patient's medical data. The AI layer then diagnoses the ailment through clever techniques. Data cleaning and imputation tasks are completed using DAEM after data collection. With the use of a deep learning architecture, missing values can be found for effective data handling and standardization. The GFO method is used to extract the most significant features from the imputed data in order to precisely diagnose diseases and categorize their types. Reducing data dimensionality also aids in reducing processing time in general. Furthermore, the disease type is classified from patient data using the state-of-the-art ColBGaN classification algorithm based on the chosen qualities or characteristics. The primary benefits of the proposed DACL model are its accuracy in disease diagnosis, applicability for low-cost healthcare sectors, and reduced computational complexity.

The following sections serve as the remainder of the parts of this paper: The thorough literature analysis in Sect. “Related works” examines several AI methods utilized in IoMT systems for disease diagnosis. The suggested DACL framework is clearly explained in “Proposed methodology” along with its flow and appropriate descriptions. Additionally, Sect. “Results and discussion” compares and validates the outcomes of the suggested DACL framework using a number of performance metrics. In Section, the overall paper is summarized together with its future scope.

## Related works

This section examines the most modern AI techniques paired with IoMT systems to identify various diseases. Additionally, it evaluates each mechanism's benefits and drawbacks in terms of performance enhancement and disease diagnosis effectiveness.

Askar et al.^26^ discussed about the protocols, and applications of the IoMT networks for healthcare applications. The practice of using IoMT technology to remotely supervise hospitals is known as telemedicine. Patients no longer need to go to a healthcare facility or physician's office each time they experience a medical problem or an upsurge in their state of health due to this kind of care. IoMT has a number of upsides, and a few of them are outlined below:

- Patients gain from better treatment alternatives involving more affordable and high-quality medical equipment and pharmaceuticals.
- In the long run, patients are going to conserve money simply as a consequence of this matter.
- Superior therapeutic results are provided.
- Because IoMT technologies expand practitioners' and researchers' capabilities, it assures an increased degree of trust in healthcare providers.
- Errors are significantly decreased.
- One can monitor and control their medication intake.
- It is easy to sustain the daily utilization of medical supplies in an IoMT system.
- It offers better prevention of disease.

The ability to sense real-time data for patient monitoring can be added to existing healthcare devices by adding gauges, signaling conversion devices, and transmission modems. IoMT components, including smart wearable, personal medical supplies, healthcare mobile uses, and POC kits, can link with medical professionals who are located remotely. They were additionally employed to manage typical medical status, promote well-being, avoid illness, assist remotely in situations of crisis, and more. Here are a few illustrations of IoMT end-user mobile applications.

1. *Disease management* The control of chronic pathological conditions like diabetes, hypertension, and coronary artery disease holds potential for IoMT-enabled devices. This device is used to keep track of all internal body functions such electrolyte levels, heart rate, weight, and irregular blood sugar readings. These devices collect vital current information, which gets processed at greater speeds and utilized to predict sickness development as well as future therapy and dose modifications. Additionally, standardized collection of data can assist researchers in understanding how specific diseases are disseminating in a specific region.
2. *Telehealth* Data from wireless devices is tracked centrally by the medical professional's practice. Healthcare management that analyzes current data with historical records and decides how to efficiently handle patients in the years to come is made possible by gathering and assessing user-specific input. This artificial intelligence helps service providers monitor, delegate directing and remote management duties to IoMT technology, lower the cost of implementing maintenance services, and make the best use of their technology. Additionally, the effectiveness of medical professionals has increased as a result of remote supervision, along with a reduction in patient abandonment rates.
3. *Efficient drug management* The IoMT-based RFID tags have been employed to track supply prices and problems with pharmaceutical availability. For instance, labeling drug wrapping enables manufacturers to guarantee their product's reliability.
4. *Online assistance* In a crisis, professionals can provide drugs and gauge a patient's response using real-time sensor data. Such quick actions reduce acute expenditures while delivering advanced healthcare services.

Juneja et al.^27^ implemented an AI integrated IoMT systems for proper patient care and disease diagnosis. The main purpose of this study is to investigate the several types of learning techniques including machine learning, deep learning, and other regression based models for IoMT systems. The Internet of Medical Things (IoMT), a modern bio-analytical platform that integrates software programs and network-connected medical gadgets for improving human health, appears in the recent period of time. Yaqoob et al.^28^ applied a modified artificial bee-colony optimization technique incorporated with a federated learning mechanism for an effective disease diagnosis in IoMT systems. A privacy-aware collaborative learning method of a common framework is what federated learning aims to achieve by retaining the information on the gadget. As a result, federated learning users will benefit from individualized machine learning and resolve issues with confidentiality. In order to improve cardiac disease prediction while resolving privacy concerns in a healthcare system, the suggested framework utilizes a hybrid method using FedMA and M-ABC optimization approaches. The main objective of this study is to decrease training time and increase communication effectiveness while increasing the accuracy of coronary artery disease prediction. Alamelu et al.^29^ introduced a hybrid lion-butterfly optimization technique for heart disease prediction in the IoMT systems. Here, the Yolo deep learning architecture is also employed to obtain the better classification performance outcomes. By including the most recent CSPDark-Net53 into the fundamental framework to attain quick modeling convergence and lower development time expenses, this study deals with the problem of learning time.

Dwivedi et al.^30^ discussed about the major applications and role of IoMT systems in healthcare applications. The authors of this work aim to analyze the benefits of adopting IoMT networks with their technological advancements. Moreover, it provides the clear view about the layered architecture of IoMT systems for improving the developments of healthcare applications. Nigar et al.^31^ developed a new chronic disease diagnosis system using machine learning technique for healthcare applications. The authors of this study aimed to analyze 6 different types of chronic diseases such as diabetes, pneumonia, heart disease, Alzheimer disease, and brain tumor. Moreover, the suggested framework comprises the main modules of data analysis, disease diagnosis and performance evaluation. The IoT devices like smart watches and mobile phones are used to obtain the medical data, which are fed to the computational system for analysis and disease diagnosis. The subsequent module performs the operations of preprocessing, augmentation, and feature analysis. The region of interest (ROI) is generated by resizing and cropping the images during preprocessing. This process shortens the computation time while improving the trained classifier's accuracy and dependability. Moreover, the performance of the suggested framework is determined according to the parameters of accuracy and loss. Yet, it required to minimize the complexity of classification with low loss rate. Yildirim et al.^32^ developed a new IoMT framework with the standard machine learning techniques for health data analysis and disease diagnosis. The system's accuracy, however, falls short of expectations, which is the study's main shortcoming.

Karthikeyini et al.^33^ applied an innovative deep learning algorithms such as GRU and stacked auto-encoder for efficiently characterizing the input data in order to accurately predict the type of disease. Deep learning is an AI method that makes use of multiple neural network layers and vast amounts of data to optimize different algorithms for a specific task. In many areas of development—from exploration to forecasting to decision-making—deep learning has shown positive potential. DL can identify patterns of particular diseases in inpatient electronic medical records and notify practitioners of any anomalies. Ausin et al.^34^ developed an IoMT paradigm for an effective heart disease management. This study aims to develop a novel heart activity monitor for calculating and monitoring the stroke volume. This instrument for the diagnosis of cardiac disease makes use of many sorts of sensor equipment. The suggested method is quite sophisticated in design, though, and takes more time to predict disease outcomes. Gou et al.^35^ developed a new and flexible cognitive medical decision system for strengthening the end-user confidence with proper training and computational performance. Zhang et al.^36^ introduced a physics guided deep learning algorithm for identifying and classifying coronary disease by adopting IoT technology. Shelk et al.^37^ investigates about the impacts of adopting IoMT systems for healthcare applications. In this study, some of the well-known machine learning algorithms including neural network, LR, NB, MLP, and etc. have been validated according to the vital signs of diseases like blood pressure and heart rate. Yet, the suggested algorithms require some more amount of time for disease prediction and prognosis. Table 1 provides the summary of recent studies in AI-IoMT systems.

**Table 1:** Literature review on AI-IoMT systems.

References	Methods	Discoveries	Fallouts
^38^	Hybrid CNN stacked group handling method	AI enabled smart healthcare for heart-stroke prognosis	Average accuracy = 98.6% and prediction rate = 96.68%
^39^	Automated Agri-Aid	It offers an immediate patient care with the use physiological features	Accuracy = 98%
^40^	SDN enabled big data model	It uses an unsupervised learning algorithms for medical disease classification	Network throughput = 90%, and drop out = 10%
^41^	DepDL with neighborhood component analysis	It aims to perform depression detection system for smart hospitals	Classification accuracy = 98.90%
^42^	Machine learning based behavior modification system	Chronic disease diagnosis and drug management in smart healthcare	Accuracy = 99%
^43^	Deep learning algorithm	Cloud enabled breast cancer detection framework	Training accuracy = 98.86% and testing accuracy = 97.81%

In this research review, it is determined that the standard IoMT-enabled healthcare applications have significant difficulties related to the following aspects:

- An extension of the disease prognostic period.
- High price.
- Weak dependability and scalability.
- Minimized disease classification and identification accuracy;
- Reduced effectiveness.

The proposed study's goal is to use AI-IoMT systems for smart healthcare to come up with a new method for diagnosing diseases.

## Proposed methodology

**Figure 1:**
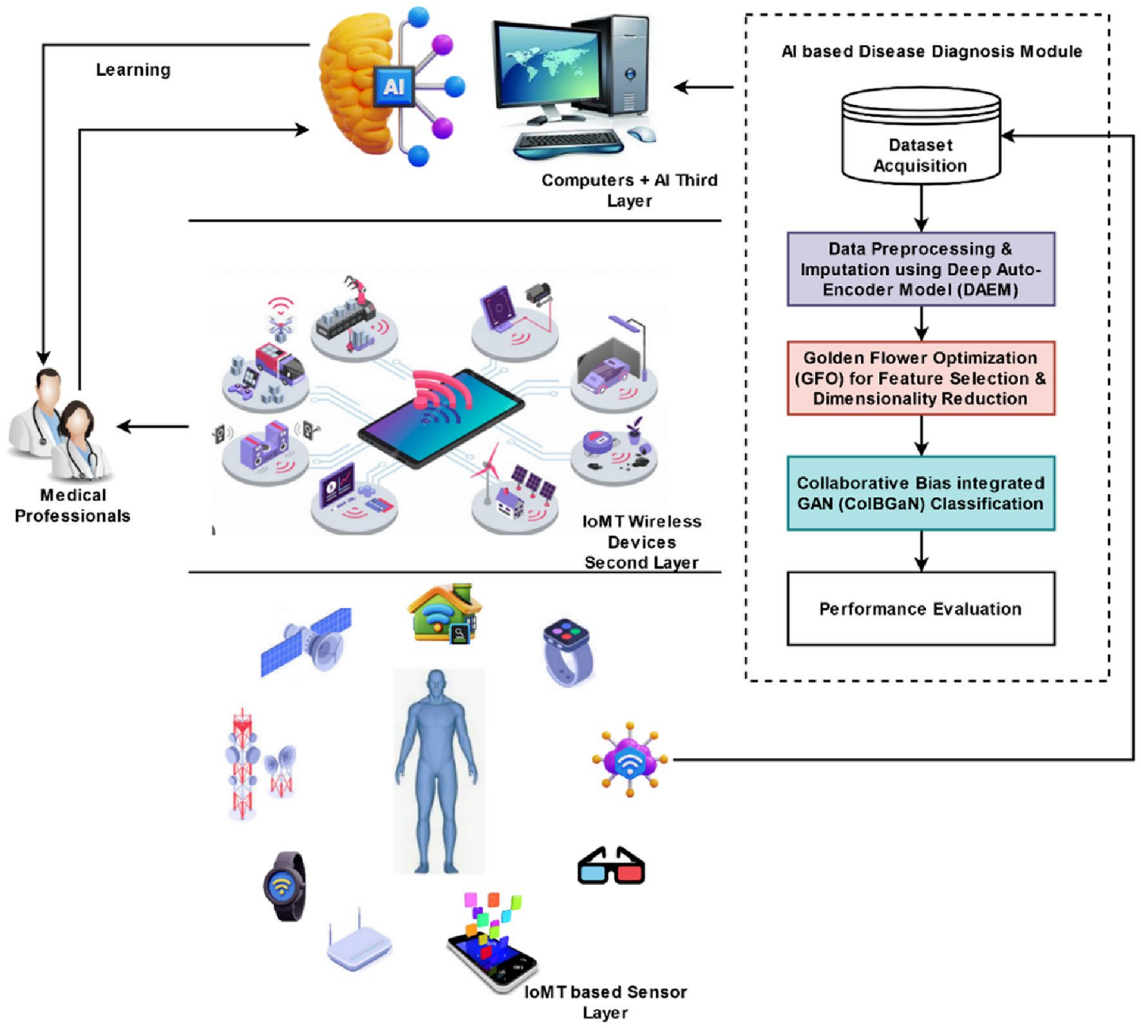
Flow of the proposed DACL model.

This section provides the complete explanation for the proposed Deep Auto-Optimized Collaborative Learning (DACL) Model for disease prognosis using AI-IoMT systems. The primary contribution of this study is the creation of an effective automated detection method that can accurately identify and categorize diabetes, heart disease, and stroke from the provided medical records. In this study, a sophisticated AI mechanism that incorporates a meta-heuristic model has been created for this aim. The suggested system's flow is depicted in Fig. 1, where theAI-IoMT systems' three distinct layers—sensor, wireless device communication, and AI computing—are presented. The following stages are included in the suggested computing paradigm as well:

- Deep Auto-Encoder Model (DAEM) based imputation
- Golden Flower Optimization (GFO) based Feature Selection
- Collaborative Bias Integrated GAN (ColBGaN) model for disease classification

First, patient medical data is gathered using sensors as part of the first tier of the proposed AI-IoMT architecture for smart healthcare. After that, using sophisticated methods, the AI layer makes the diagnosis. After data collection, DAEM is used to finish activities like data cleaning and imputation. Missing values can be detected using a deep learning architecture for efficient data handling and standardization. For precise diagnosis and classify diseases, the most significant characteristics are determined from the imputed data using the GFO approach. For instance, processing time can be reduced through decreasing the size of the data. The most sophisticated ColBGaN classification algorithm is also used to extract the illness type from patient data based on any characteristics or variables that have been selected. The main advantages of the proposed DACL model are its reduced computing cost, adaptability for sectors of affordable healthcare, and predictability in identifying diseases. The role of the DAEM in data imputation is acknowledged but not elaborated upon. Detailed methodology including techniques used for data imputation, along with validation of the imputed data’s accuracy and reliability, would lend credibility and robustness to your framework. At this stage, the imputed data (i.e., normalized medical data record) is gathered for feature selection and dimensionality reduction. Since the GFS technique offers the best and most optimal way to choose the most significant characteristics from the available data, it is employed in this study to accomplish this purpose. The primary objective of implementing this method is to minimize the total processing time required for the classification of disease prognosis. Furthermore, it aids in accelerating the classifier's speed while determining and categorizing the illness kind from medical data. The ColBGaN categorization method is employed to offer a precise prognosis for the condition. It is a state-of-the-art hybrid deep learning system designed with disease identification and classification in mind. Current research often use a variety of deep learning algorithms for disease identification and classification. Still, most methods suffer from specific challenges such overfitting, poor forecasts, increased testing and training complexity, and decreased accuracy. Using a novel classification mechanism known as the ColBGaN model, the proposed study aims to efficiently identify and classify the kind of illness from the patient medical information. In this work, the loss function is tuned to its optimal value using the WDO technique. Optimizing classifier performance for increased accuracy is the aim of hyper-parameter tweaking. The loss function is important because the deep learning technique is used to simplify the classification process with a low error value.

### Data imputation

**Figure 2:**
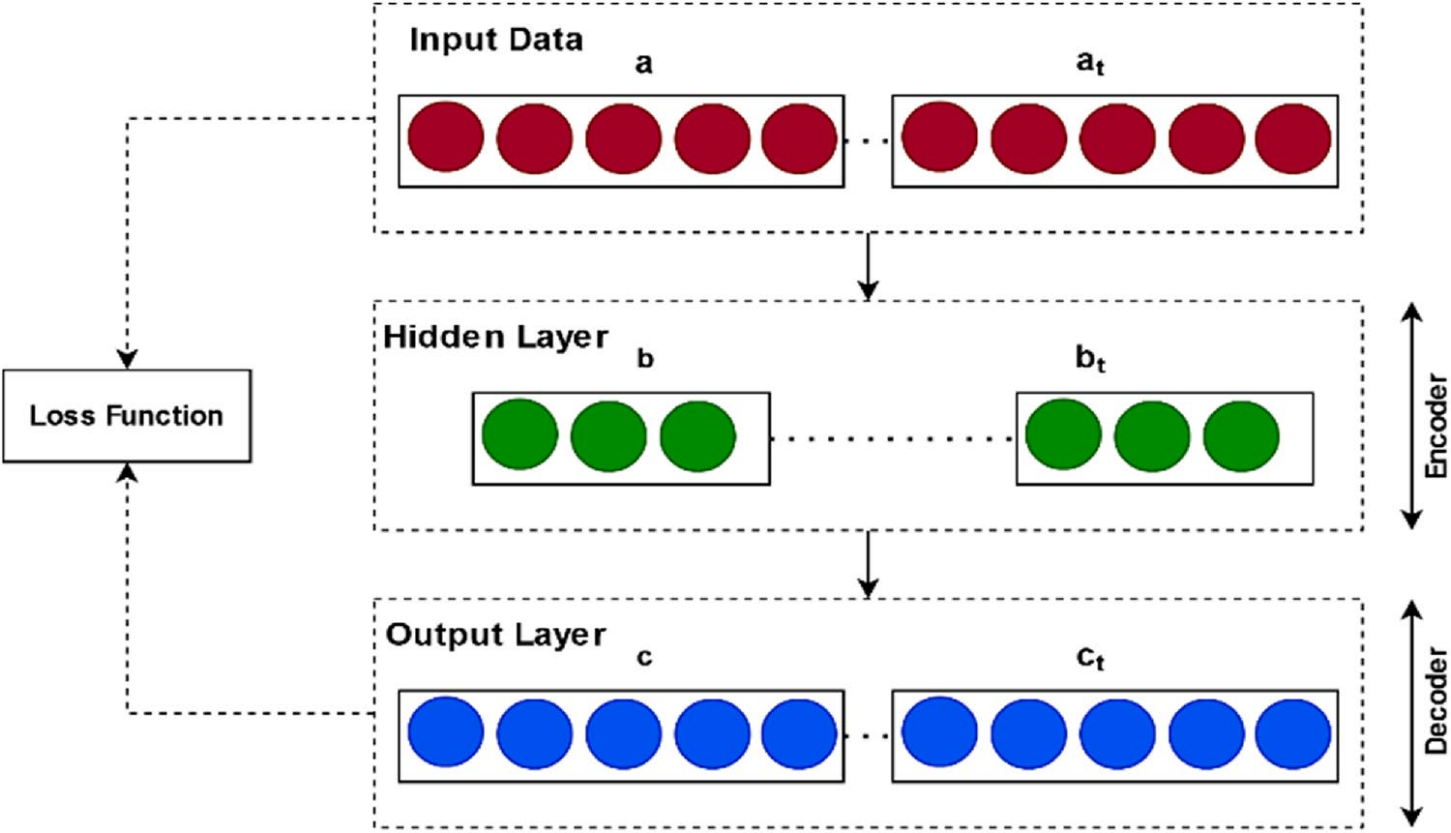
Architecture model of DAEM.

Substantial missing data in essential functions of the program might result in unreliable predictions and erroneous estimations. By causing incorrect presumptions, it might obstruct learning. To comprehend the impact of data that is missing on a specific analysis or way to analyze missing data, it is vital to understand the mechanics of missing information. After valuable data is eliminated the deletion technique has various drawbacks, including a loss of accuracy and outcome bias. Prior to using the normal complete information look at on the filled data, the missing data imputation approach connects the missing information. However, several of these imputation techniques lack the ability to manage outliers and are unable to handle data gaps with multi-type factors, such as binary, categorical, and continuous variable blends. In the meantime, a number of methods based on deep learning are being established recently to address this problem. But, the traditional deep learning models frequently have a number of issues, including poor training effectiveness, intricate networks, local minimums, problematic control parameter tuning, and a gradient loss. Therefore, the proposed study aims to implement a novel deep learning mechanism, named as, DAEM for data imputation and missing information handling. It is an unsupervised learning strategy that makes use of a network of neurons to learn an efficient information representation or encoding in order to recreate the initial input information. The architecture model of the proposed DAEM shown in Fig. 2. Since, it is an unsupervised learning technique that reconstructs the original input data by using a neural network to learn optimal data representation or encoding. This function is defined as the cross entropy for discrete data and as the mean squared error for continuous data. It lowers the squared error across the input and the output in our imputation process. To this end, the squared error present in the majority of conventional auto-encoders is intended. It might be more reasonable in this case to forego using nonlinearity squashing in the decoder due to the Gaussian determination.

The proposed DAEM architecture comprises two major components such as encoder and decoder, where the mapping function of the encoder is used to transfer the input vector as represented in the following model:

1 $$ b = \varepsilon_{\theta } \left( a \right) = \sigma \left( {\omega a + \beta } \right) $$

where the input vector $a \in {{\mathfrak{A}}}^{x}$ is transferred to the hidden layer in the form of $b \in {{\mathfrak{A}}}^{y}$. The value of $\theta = \left\{ {\omega ,\beta } \right\}$, the weight matrix $\omega$ comprises the rows and columns of $x \times y$, $\beta$ indicates the bias vector, $\sigma$ is the sigmoid function and $\varepsilon$ denotes the encoder function. Consequently, the decoding unit ${{\mathcal{D}}}$ receives the hidden representation $b$ for performing mapping operation according to the reconstructed vector $c \in {{\mathfrak{A}}}^{x}$ as represented in the following equation:

2 $$ c = {{\mathcal{D}}}_{\theta \prime } \left( b \right) = \sigma \prime \left( {\omega \prime a + \beta \prime } \right) $$

where the value of $\theta \prime = \left\{ {\omega \prime ,\beta \prime } \right\}$, and the weight matrix $\omega \prime$ comprises the rows and columns of $x \times y$. Consequently, the obtained parameters $\theta$ and $\theta \prime$ are used to reduce the reconstruction error, which are defined as shown in the following model:

3 $$ \theta^{*} \theta \prime * = argmin\frac{1}{x}\mathop \sum \limits_{i = 1}^{x} \ell \left( {a^{\left( i \right)} ,b^{\left( i \right)} } \right) = argmin\frac{1}{x}\mathop \sum \limits_{i = 1}^{x} \ell \left( {a^{\left( i \right)} ,{{\mathcal{D}}}_{{\theta^{\prime}}} \left( {\varepsilon_{\theta } \left( {b^{\left( i \right)} } \right)} \right)} \right) $$

Reconstruction losses among input and output data, including MSE loss, can be reduced via the auto-encoder. The objective of the auto-encoder is to rebuild the output with the least amount of function loss possible. Based on these operations, the missing fields of information are identified and imputed in the proposed system. After data collection, data preparation is carried out to enhance the quality of the data. Feature elimination, or the removal of pointless features, is typically a step in the preliminary processing of data. By using encoding, information pertaining to categories is subsequently transformed into numeric form. The benefit is that because each category is shown as only one input, the problem space's proportions remain unchanged. To ensure that all scaled features have the same effect, data normalization modifies the value of a feature in accordance with predetermined principles. In order to enhance data quality, it can also be used to get rid of data outliers.

### Golden flower search (GFS) optimization for feature selection

For the purposes of feature selection and dimensionality reduction, the imputed data (i.e., normalized medical data record) is collected at this stage^44,45^. Since the GFS technique offers the best and most optimal way to choose the most significant characteristics from the available data, it is employed in this study to accomplish this purpose. The primary objective of implementing this method is to minimize the total processing time required for the classification of disease prognosis. Furthermore, it aids in accelerating the classifier's speed while determining and categorizing the illness kind from medical data. Over the past few decades, there has been a tremendous growth in several meta-heuristic techniques. These tactics combine swarm cognition and intelligent computation, drawing inspiration from the biological behavior of many creatures. The recent studies employ a variety of meta-heuristic models for feature selection, however most of these techniques suffer from problems with reduced efficiency, challenging searching, and processing overhead. The goal of the proposed work is to implement a new and cutting-edge GFS technique for feature minimization. Furthermore, the efficacious, adaptable, and user-friendly nature of the suggested GFS technique are its main advantages. The flower pollination method is an optimization strategy based on swarms and inspired by the behavior of flowers. Flowers multiply through a process called pollination, in which pollinators move pollen from one bloom to another. Generally speaking, there are two types of pollination: abiotic and nomic. During the abiotic pollination process, which does not require the presence of live organisms like birds, insects, or other species that serve as pollinators, pollen is dispersed by blowing or diffusion.

**Figure 3:**
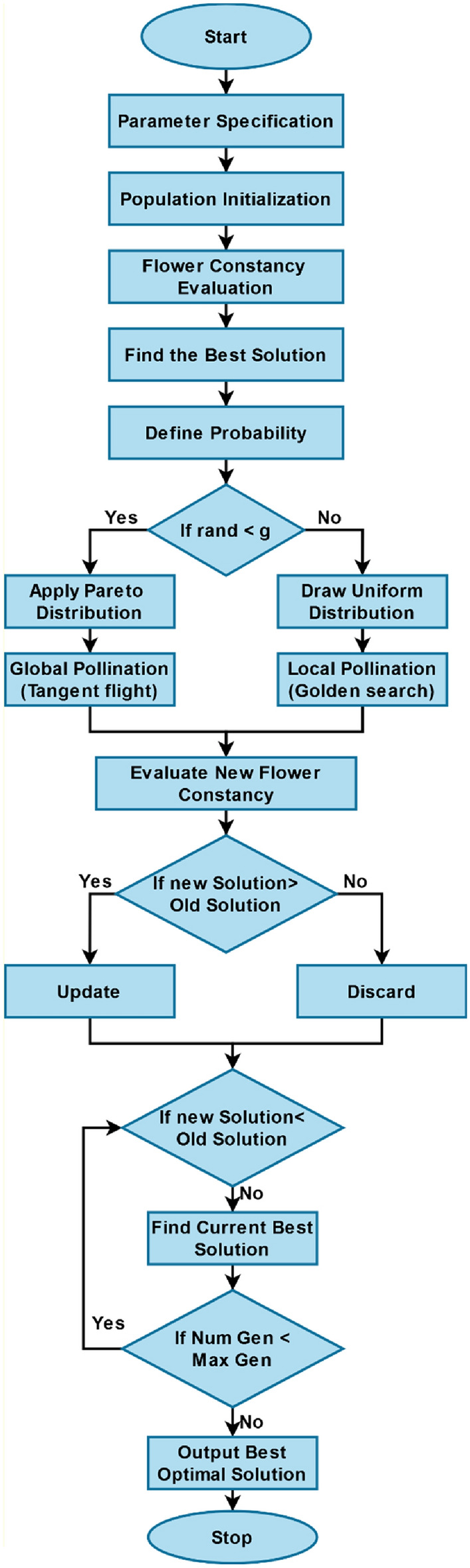
Flow of GFS optimization technique.

A flower is deemed to be self-pollinating if it reproduces using either its own pollen or the pollen from other flowers of other blooms on the exact same plant. Cross-pollination occurs when pollinators move pollen over significant distances. Local pollination, also known as self-pollination, is different from global pollination, which refers to pollination that takes place across an extensive region. This method is employed to complete global pollination due to the pollen's greater motions and Levy flying tendency. In this optimization algorithm, the local pollination is performed with the golden search, and the global pollination process is performed with the tangent flight operation. Instead of using Lévy flights, as is done in the global explorative phase of the GFS technique, we have modified the algorithm and employed a substitute heavy-tailed likelihood distribution function. At first, the solution vector is formed as represented in the following equation:

4 $$ G_{h + 1} = G_{h} + {\mathcalligra{s}} \times S\left( g \right) \times \left( {G_{h} - \tau *} \right) $$

where $S$ indicates the pareto distribution, $G_{h + 1}$ is the solution vector with iterations $h + 1$, $\tau *$ represents the global best solution, and ${\mathcalligra{s}}$ represents the step size. The local and global searching methods for flower pollination are under management of the switching probability. Pollens can research and be employed throughout both global and local pollination processes owing to the switching probability. To safeguard the diversity of the techniques, pollens have the right to investigate the critical region in order to determine the ideal strategy for global pollination. The flower pollination method can handle complex scenarios and operates more effectively than alternatives due to its exploration and operation stages. Then, the golden ratio and inverse golden ratio are computed, and the obtained solution vectors ${{\mathcal{V}}}_{1}$ and ${{\mathcal{V}}}_{2}$ are defined as shown in below:

5 $$ {{\mathcal{V}}}_{1} = m + 0.0618\left( {n - m} \right) $$

6 $$ {{\mathcal{V}}}_{2} = n - 0.0618\left( {n - m} \right) $$

where $m$ and $n$ are the minor and major segments. Consequently, the tangent function is also estimated with the use of tangent flight model. This function offers a great amount of exploration potential due to its frequency and alternation between − infinity and + infinity, allowing for an ideal balance between exploration and amplification. A global step of the form “${\mathcalligra{s}}$” in which the tangent function operates identical to the Levy flight function, drives the equation for movement in the tangent approach. This piece simply refers to it as a tangent flight as calculated in below:

7 $$ G_{h + 1} = L + \left( {U - L} \right) \times rand\left( X \right) $$

where $L$ and $U$ are the lower and upper bound values, and $X$ denotes the size of problem. This algorithm commences by generating a random beginning population within the solution space, much like several other population-based optimization methods. The following equation is used to dissipate the initial solution uniformly across the search space.

8 $$ G_{h + 1} = G_{h} + {\mathcalligra{s}} \times tan\theta \times \left( {G_{h} - \tau *} \right) $$

where $h$ is the iteration, ${\mathcalligra{s}}$ represents the step size, and $\tau *$ denotes the global best solution. The method is used to find the best solution, which is then used to select the most important features from the imputed data. The overall optimization process is represented in Fig. 3.

### Disease prognosis using ColBGaN

The ColBGaN classification approach is utilized to deliver an accurate sickness prognosis after feature selection. This state-of-the-art hybrid deep learning method was created especially for the diagnosis and categorization of illnesses. In most contemporary studies, a variety of deep learning algorithms are employed to classify and identify disorders. But the main challenges faced by most techniques include overfitting, poor forecasting, greater complexity in training and testing, and decreased accuracy. The proposed work aims to use a novel classification mechanism known as the ColBGaN model to efficiently identify and classify the kind of illness from the patient medical information. These are the main components of the suggested classifier:

- Generator
- Discriminator
- Classifier

To make the created information more similar to the actual information and to forecast the distribution of actual data, the data generator trains its output using adversarial feature discrimination and classification units. This is more than just using the source data to extract features. The ColBGaN classifier uses two different feature extractors to distribute the feature weights of the produced and original data after first adding arbitrary labels. In order to ultimately correct the distribution bias pattern in unbalanced learning and to stop the generated data from being instantly categorized into the majority groupings. The two cooperative adversarial training stages of the suggested ColBGaN classifier—the generator-classifier and the data generator-discriminator, respectively—are used in its conception and construction. An extra classifier connected to the data generator can produce a fresh adversarial training.

In this model, the dimensions of $\alpha_{t}$ and $\beta_{t}$ are combined using the addition operation so that they can be mapped into an innovative contingent feature vector $\gamma_{t}$, for more information enhancement. The operation is clearly described by using the following mathematical models:

9 $$ \alpha_{t} = \sigma \left( {\alpha ;{\mathcalligra{w}}_{\alpha } } \right) $$

10 $$ \beta_{t} = \varphi \left( {\beta ;{\mathcalligra{w}}_{\beta } } \right) $$

11 $$ \gamma_{t} = \alpha_{t} + \beta_{t} $$

where $\beta \in M$ denotes the data label, $\gamma \sim {{\mathcal{N}}}\left( {0,{{\mathcal{I}}}} \right)$ defines the noise, $\sigma \left( * \right)$ and $\varphi \left( * \right)$ are the feature embedding functions that are applied with the use of fully connected network layers. Then, the $\gamma_{t}$ is passed to the encoding unit for constructing an augmented data as represented in the following model:

12 $$ a\prime = Encoder\left( {\gamma_{t} ;{\mathcalligra{w}}_{e} } \right) $$

where ${\mathcalligra{w}}_{e}$ represents the parameter for encoding function. In this instance, $\beta$ and $\gamma$ are considered as the input parameters that are used to formulate the conditional generator. Then, the obtained data $a\prime$ is controlled with the information of input $\beta$, which indicates that every label's adoption is crucial for producing the right data for minority classes during the training operation. The output of the feature discriminator ${\mathbb{D}}{{\mathfrak{d}}}\left( * \right)$ constitutes a scalar that distinguishes the input from generated information derived from actual data. In tandem, the data generator ${{\mathcal{G}}{\mathfrak{y}}}\left( * \right)$ and feature discriminator ${\mathbb{D}}{{\mathfrak{d}}}\left( * \right)$ acquire the traits and increase the likelihood that ${\mathbb{D}}{{\mathfrak{d}}}\left( * \right)$ would err and conclude that the input came from the original data and not from ${{\mathcal{G}}{\mathfrak{y}}}\left( * \right)$. The following equation represent this process:

13 $$ \begin{array}{*{20}c} {min} \\ {{{\mathcal{G}}{\mathfrak{y}}}} \\ \end{array} \begin{array}{*{20}c} {max} \\ {{\mathbb{D}}{{\mathfrak{d}}}} \\ \end{array} {\mathbb{E}}_{a\sim k\left( a \right)} \left[ {{\mathbb{D}}{{\mathfrak{d}}}\left( a \right)} \right] + {\mathbb{E}}_{{\left( {\beta ,\gamma } \right)\sim k\left( {\beta ,\gamma } \right)}} \left[ {1 - {\mathbb{D}}{{\mathfrak{d}}}(\left( {{{\mathcal{G}}{\mathfrak{y}}}\left( {\beta ,\gamma } \right)} \right)} \right] $$

where ${\mathbb{E}}_{a\sim k\left( a \right)} \left[ {{\mathbb{D}}{{\mathfrak{d}}}\left( a \right)} \right]$ defines the expectation of the discriminator, and $k\left( a \right)$ indicates the distribution value. Moreover, the loss function is computed according to the following model:

14 $$ {\mathbb{L}}_{{{{\mathcal{G}}{\mathfrak{y}}}}} \left( {\alpha ,\beta } \right) = Entropy({\mathbb{D}}{{\mathfrak{d}}}\left( {{{\mathcal{G}}{\mathfrak{y}}}\left( {\alpha ,\beta } \right),1} \right) + \delta $$

where $Entropy()$ indicates the entropy loss function, and $\delta$ represents the optimal parameter computed by using the WDO technique. With the help of classifier, the disease is accurately detected and categorized with the appropriate class.

### Hyper-parameter tuning based WDO

In this work, the loss function is tuned to its optimal value using the WDO technique. Optimising classifier performance for increased accuracy is the aim of hyper-parameter tweaking. The loss function is important because the deep learning technique is used to simplify the classification process with a low error value. Typically, the proposed WDO technique is inspired by the riverbed water circulation concept, in which each particle acts independently as an autonomous agent that starts moving at a given velocity and baseline material value. Every drop that moves will remove soil from the substrate of the path based on its speed. Since droplets of water tend to gravitate towards the river with the least amount of soil, the shortest and best path is the one with the least quantity of soil. This method requires that the amount of dirt on each graph border—a user-selected value empirically derived based on a specific application—be set beforehand in order to solve the given optimal problem. Using this method significantly improves the classifier's accuracy while lowering the error rate. The list of symbols used in this algorithm are presented with their appropriate definitions in Table 2.

**Table 2:** List of symbols and descriptions.

Parameters	Values
$i,j$	Nodes
$\zeta$	Initial quantity of soil
${\mathfrak{B}}\left( {K^{r} } \right)$,	Best solution
$Mx_{itr}$	Maximum number of iterations
$itr$	Current iteration
${\mathfrak{m}}_{x}$	Total number of water drops
$p_{u} ,q_{u} ,o_{u}$	Velocity updating parameters
$\varphi^{{{\mathfrak{m}}_{y} }}$	Global parameter
$\tau^{y}$	Soil updating parameter
$\Im^{{\mathfrak{m}}} \left( v \right)$	Specific velocity
$g_{i}^{{\mathfrak{m}}}$	Possible solution path
$\Im^{{\mathfrak{m}}} \left( {v + 1} \right)$	Updated velocity
$T$	Time
$B^{P}$	Best solution path
$best\left( . \right)$	Optimal best fitness function

The following model is used to represent any two nodes that have soil between them:

15 $$ \zeta_{i,j} = initial^{\zeta } $$

Each water drop can be transported in this structure from the current node i to the following node j in the desired region. The following model illustrates how a water drop stores an inventory of seen nodes in order to determine the potential solution path in the network:

16 $$ g_{i}^{{{\mathfrak{m}}}} \left( j \right) = \frac{{Fit\left( {soil\left( {i,j} \right)} \right)}}{{\mathop \sum \nolimits_{{{{\mathfrak{m}}} \subseteq \nu \left( {{\mathfrak{m}}} \right)}} \left( {Fit\left( {soil\left( {i,{{\mathfrak{m}}}} \right)} \right)} \right)}} $$

Additionally, each drop's transition updates the dynamic settings. Afterward, the velocity update process is illustrated in the model as shown in below:

17 $$ \Im^{{{\mathfrak{m}}}} \left( {v + 1} \right) = \Im^{{{\mathfrak{m}}}} \left( l \right) + \frac{{p_{u} }}{{q_{u} + o_{u} \ldots \zeta^{2} \left( {i,j} \right)}} $$

As a result, the following function is used to calculate the quantity of soil:

18 $$ \Delta \zeta \left( {i,j} \right) = \frac{{p_{u} }}{{q_{u} + o_{u} \times T^{2} (i,j;\Im^{{{\mathfrak{m}}}} \left( {v + 1)} \right)}} $$

Additionally, as shown in the following model, the soil of the route has been modified among nodes i and j:

19 $$ soil \left( {i, j} \right) = \left( {1 - g_{y} } \right) \times \zeta \left( {i,j} \right) - g_{y} \times \Delta \zeta \left( {i, j} \right) $$

The best path offering an optimal fitness value is chosen at the final stage of iteration, and the most effective path is determined using the following equation:

20 $$ B^{P} = arg\begin{array}{*{20}c} {max} \\ {\forall B^{{{\mathfrak{m}}}} } \\ \end{array} best\left( {B^{{{\mathfrak{m}}}} } \right) $$

As a result, the best current solution is updated for the chosen paths on the soil, as illustrated below:

21 $$ \zeta \left( {i,j} \right) = \left( {1 + g_{{{\mathfrak{m}}}} } \right) \times \zeta \left( {i,j} \right) $$

The output from this process is the best solution, which is used to calculate the ideal value for fine-tuning the loss function.

## Results and discussion

By utilizing the available datasets and a number of evaluation metrics, this part evaluates the effectiveness and performance of the suggested DACL framework. The major goal of this work is to create an effective and automated AI-IoMT system for classifying the various chronic diseases using patient medical records. Some of the well-known and open-source datasets^46,47^, including the Kaggle heart disease (Table 3), Stroke dataset, diabetes disease dataset and Statlog dataset, were used to assess the efficacy and room for improvement of the proposed DACL model. To statistically assess the proposed framework, well-known and significant performance evaluation standards including Accuracy, Precision, Recall, F1-score, sensitivity, specificity, and fall-out rate have been utilized in this study. The measures are estimated by using the following equations:

22 $$ Accuracy = \frac{TP + TN}{{TP + TN + FP + FN}} $$

23 $$ Precision = \frac{TP}{{TP + FP}} $$

24 $$ Recall or sensitivity = \frac{TP}{{TP + FN}} $$

25 $$ Specificity = \frac{TN}{{TN + FP}} $$

26 $$ F1 - score = \frac{2 \times Precision \times Recall}{{Precision + Recall}} $$

where TP—true positives, TN—true negatives, FP—false positives, and FN—false negatives. Moreover, the dataset descriptions are given below:

**Table 3:** Kaggle dataset.

Attributes	Descriptions
ID	Unique patient identity
Gender	Male or Female
Age	Number of years
Hypertension	Yes or no (1 or 0)
Heart disease	Yes or no (1 or 0)
Marital status	Yes or no (1 or 0)
Type of work	Nature of job
Location	Patients’ residential area
Average glucose level	Glucose content level in blood
Body Mass Index	Patients’ BMI value
Smoking habit	Status
Stroke	Status (1 or 0)

Another performance metric pertains to the AUC. It is determined by comparing the true positive rate versus the false positive rate at various classification levels using a ROC (receiver operating characteristic) curve. When the classifier can accurately discriminate amongst all of the positive and negative classes, it is said to have an AUC of 1. The classifier will nevertheless forecast each of the negatives as positives and inversely if the AUC is 0.

**Figure 4:**
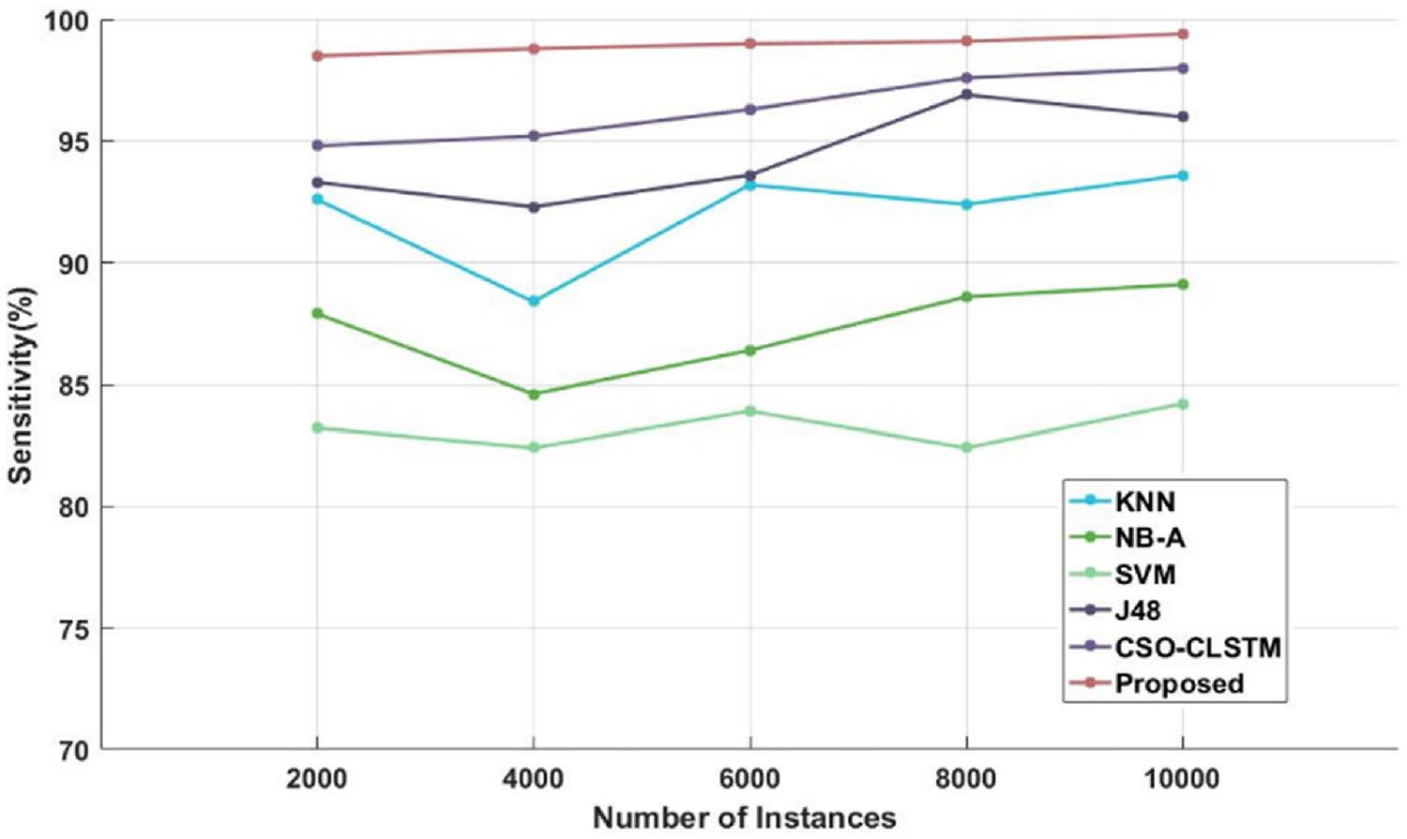
Sensitivity analysis using kaggle heart disease dataset.

**Figure 5:**
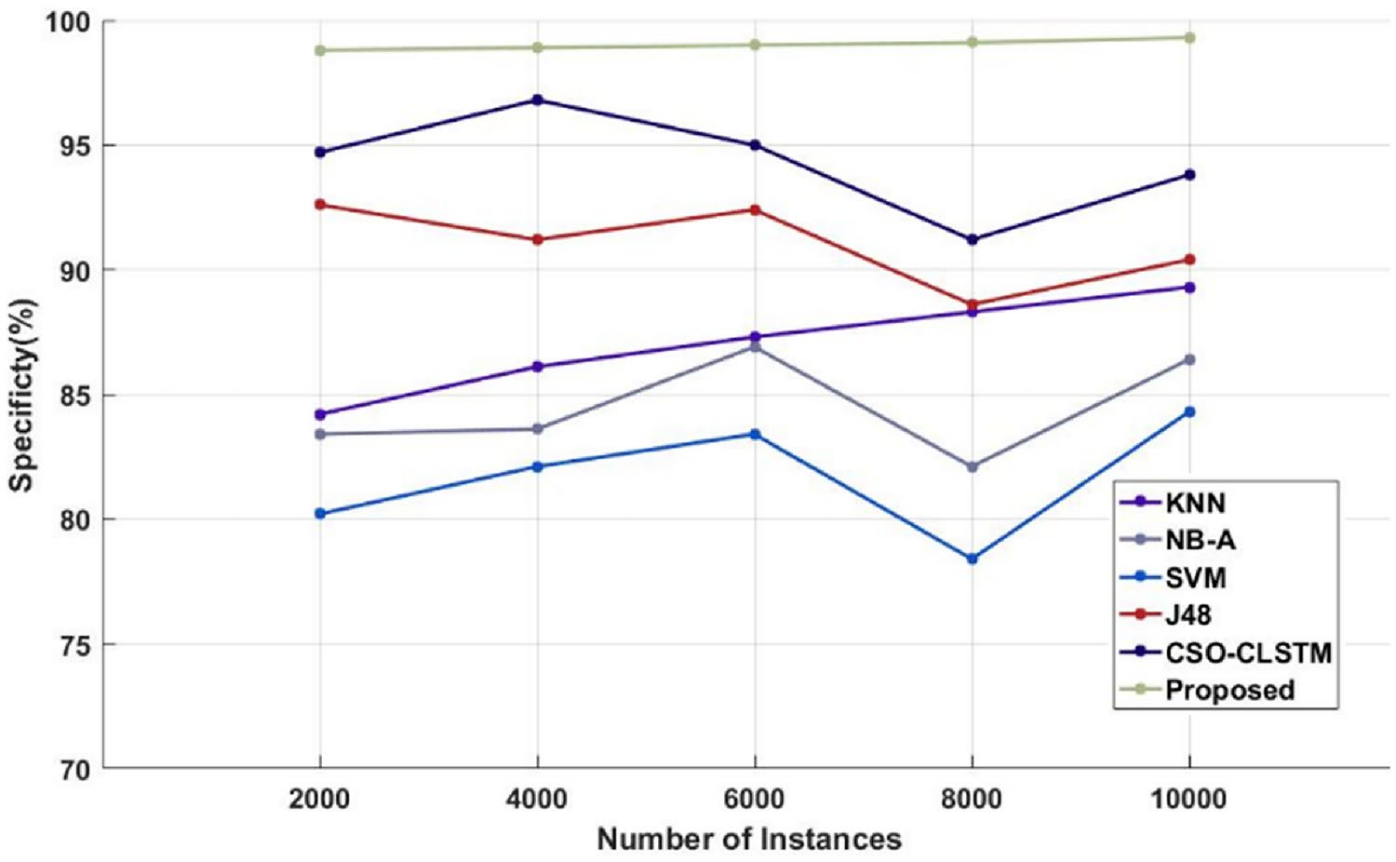
Specificity analysis using kaggle heart disease dataset.

**Figure 6:**
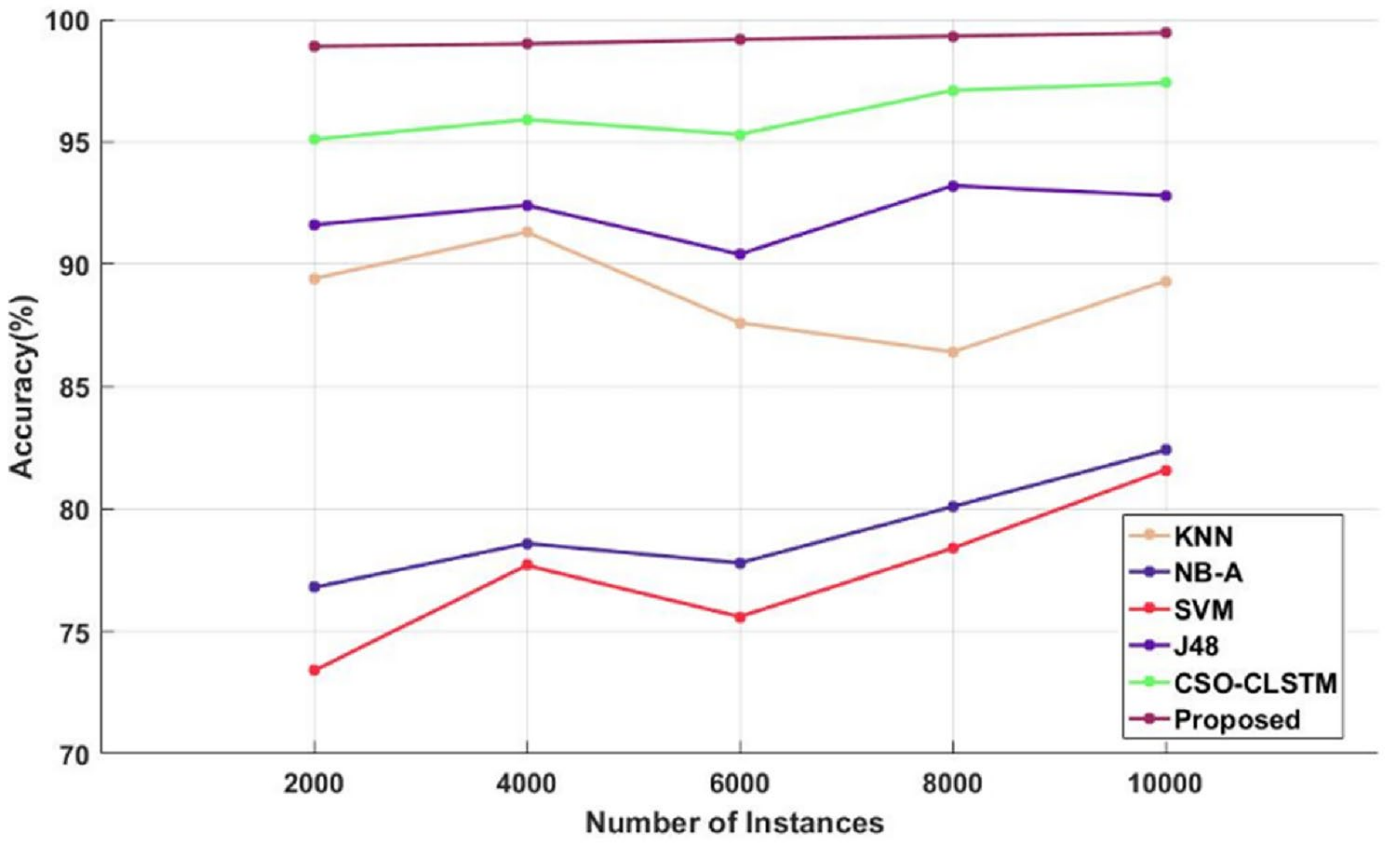
Accuracy analysis using kaggle heart disease dataset.

Figure 4 and Table 4 compare the sensitivity of the traditional^48^ and proposed illness diagnosis approaches used for healthcare applications with respect to different numbers of instances of the Kaggle disease dataset. Sensitivity, specificity, and accuracy are commonly thought to be the most crucial factors in determining how effective the categorization algorithms are. Higher system performance is indicated by these measures' higher values. As seen in Fig. 5 and Table 5, in order to compare the specificity values of the proposed and current classification systems. Figure 6 and Table 6 present a comparison of the accuracy of the traditional and proposed models using the Kaggle heart disease dataset. The results of the analysis conducted with respect to these factors clearly show that the suggested DACL model performed better.

**Table 4:** Comparison based on sensitivity using Kaggle heart disease dataset.

No of instances	KNN	NB-A	SVM	J48	CSO-LSTM	Proposed
2000	92.6	87.90	83.20	93.30	94.80	98.5
4000	88.4	84.60	82.40	92.30	95.201	98.8
6000	93.20	96.40	83.90	93.60	96.30	99
8000	92.40	88.60	82.40	96.90	97.60	99.1
10,000	93.60	89.10	84.20	96	98	99.4

**Table 5:** Comparison based on specificity using Kaggle heart disease dataset.

No of instances	KNN	NB-A	SVM	J48	CSO-LSTM	Proposed
2000	84.20	83.40	80.20	92.60	94.70	98.8
4000	86.10	83.60	82.10	91.20	96.80	98.9
6000	87.30	86.90	83.40	92.40	95	99
8000	88.30	82.10	78.40	88.60	91.20	99.1
10,000	89.30	86.40	84.30	90.40	93.80	99.3

**Table 6:** Comparison based on accuracy using Kaggle heart disease dataset.

No of instances	KNN	NB-A	SVM	J48	CSO-LSTM	Proposed
2000	89.40	76.80	73.40	91.60	95.10	98.9
4000	91.30	78.60	77.70	92.40	95.90	99
6000	87.60	77.80	75.60	90.40	95.30	99.18
8000	86.40	80.10	78.40	93.20	97.10	99.32
10,000	89.30	89.30	81.60	92.80	97.40	99.45

**Figure 7:**
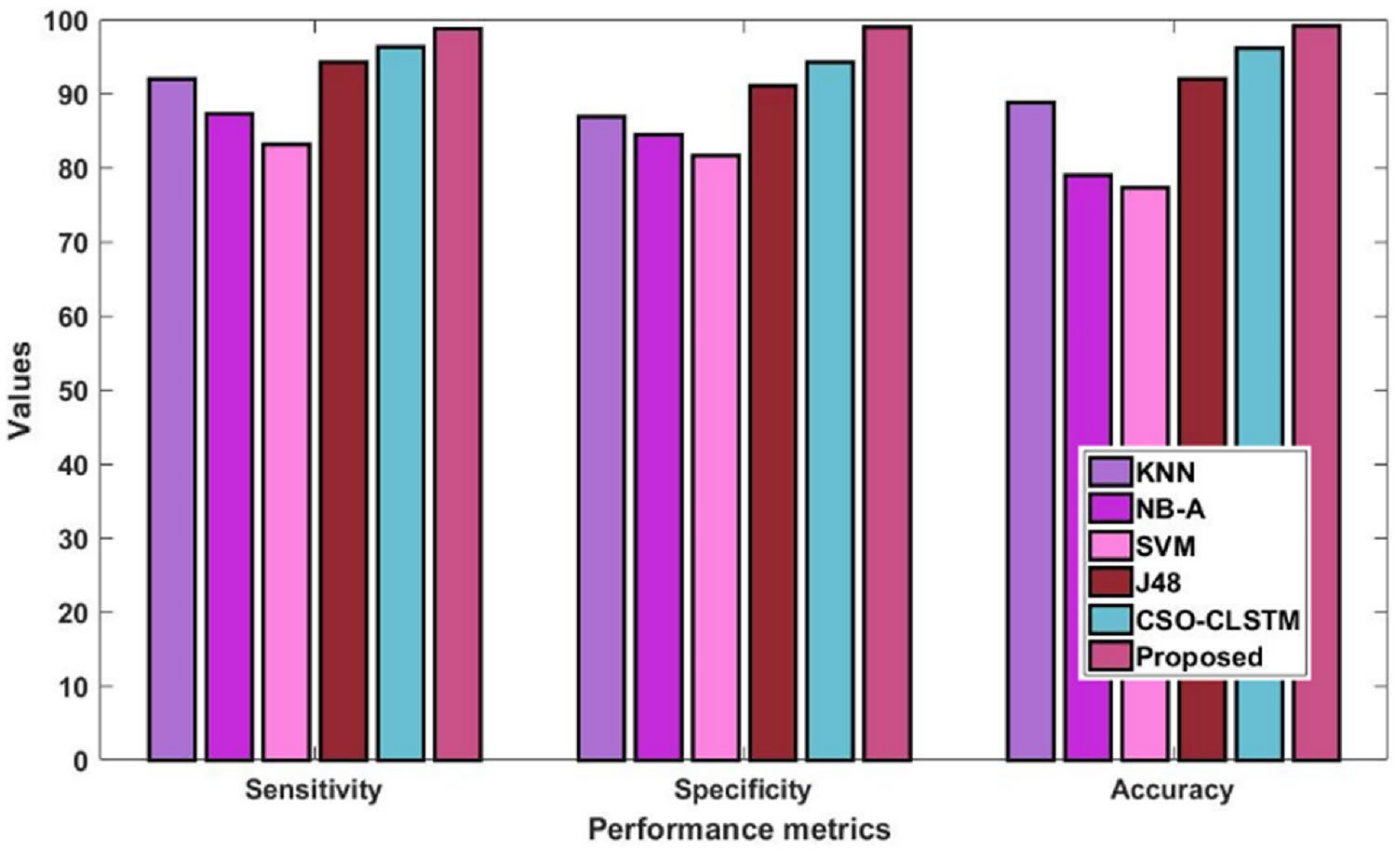
Overall comparative analysis using kaggle heart disease dataset.

Furthermore, Fig. 7 and Table 7 assess and contrast the overall sensitivity, specificity, and accuracy values of the new and classic classification methods using the Kaggle heart disease dataset. The estimated findings indicate that the suggested DACL model delivers improved performance values in comparison to earlier methods. Since several intelligence techniques are used in the data imputation, feature reduction, parameter adjustment, and classification stages of the suggested DACL. According to the accuracy analysis, compared to other conventional methods, the SVM framework performed ineffectively. Additionally, the NB-A scheme outperformed SVM in producing middling accuracy. Simultaneously, the KNN and J48 methods yielded somewhat improved accuracy. In order to achieve heavy classification, the anticipated CSO-CLSTM approach obtained an ideal value. The CSO-CLSTM model, for instance, reached a maximum accuracy of 95.10% over 2000 cases, whereas the KNN, NB-A, SVM, and J48 models only achieved limited accuracies. Using the heart disease dataset, these processes allow for an accurate diagnosis of the problem, leading to improved results with 99.2% accuracy, 98.8% sensitivity, and 99% specificity. The proposed model beat other compared approaches, as seen by the figure, which shows an improved average performance of up to 99%.

**Table 7:** Performance comparative analysis using Kaggle heart disease dataset.

Measures	KNN	NB-A	SVM	J48	CSO-LSTM	Proposed
Sensitivity	92.04	87.32	83.22	94.42	96.38	98.8
Specificity	87.04	84.48	84.48	91.04	94.30	99
Accuracy	88.80	79.14	79.14	92.08	96.16	99.2

**Figure 8:**
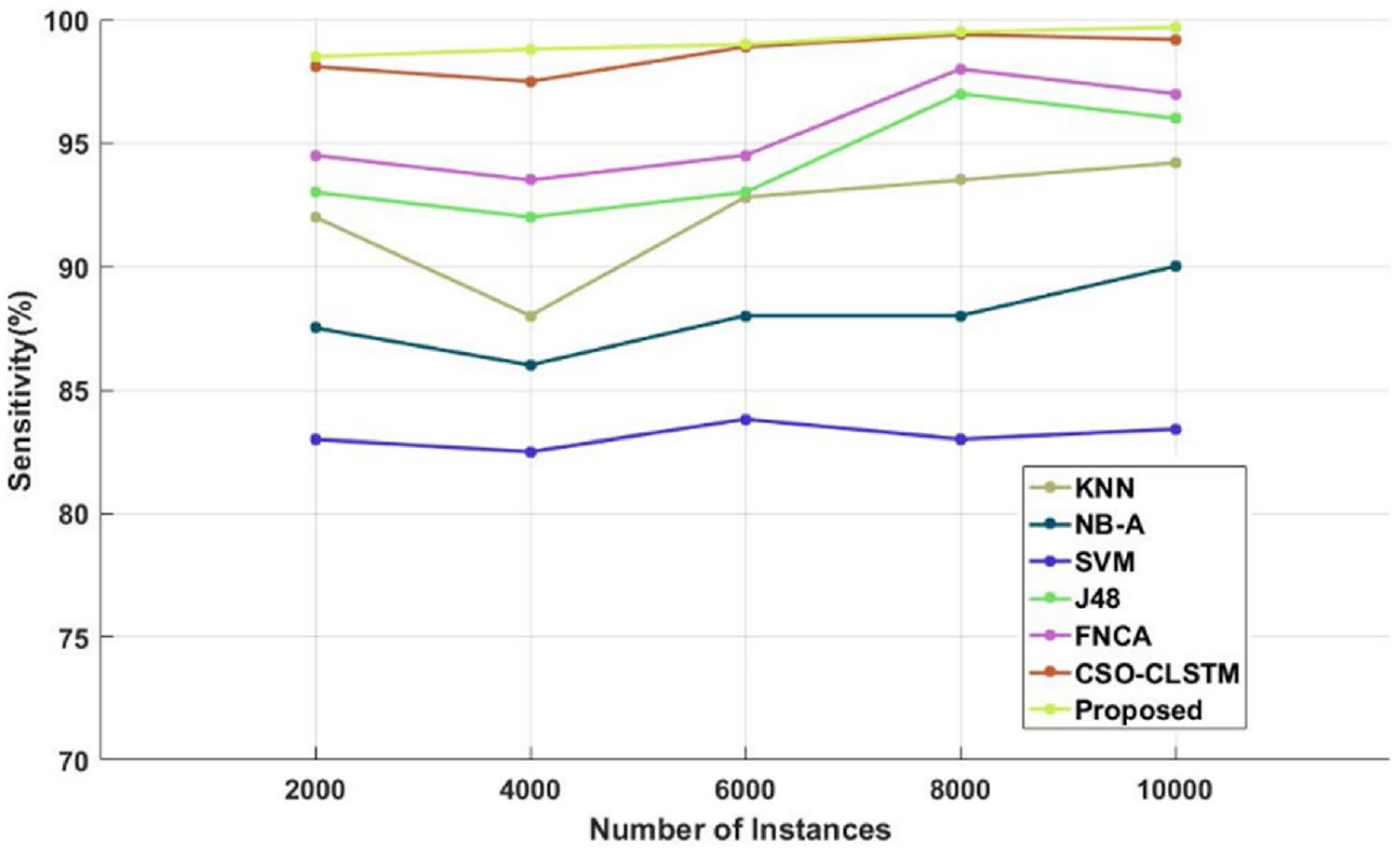
Sensitivity analysis using diabetes disease dataset.

**Figure 9:**
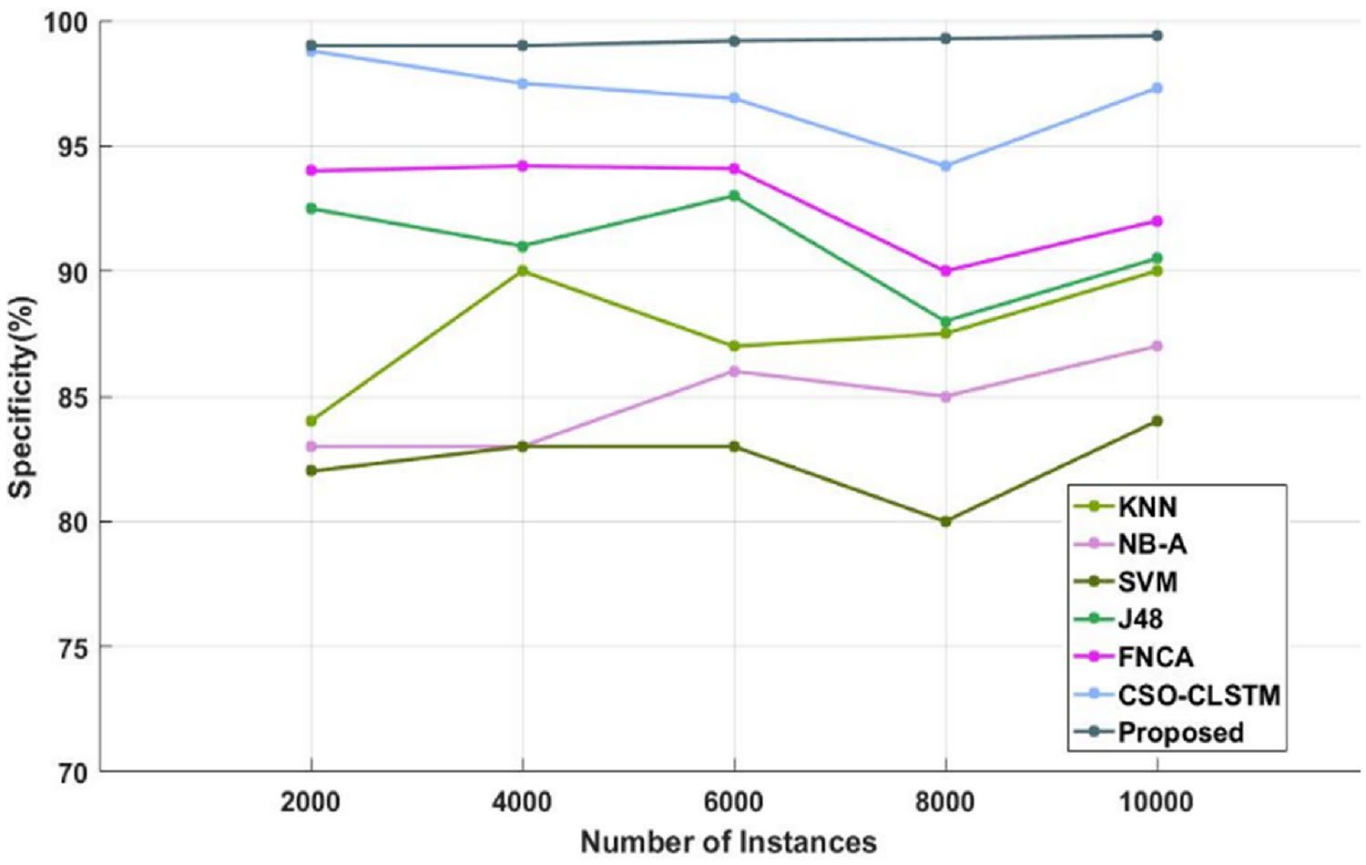
Specificity analysis using diabetes disease dataset.

**Figure 10:**
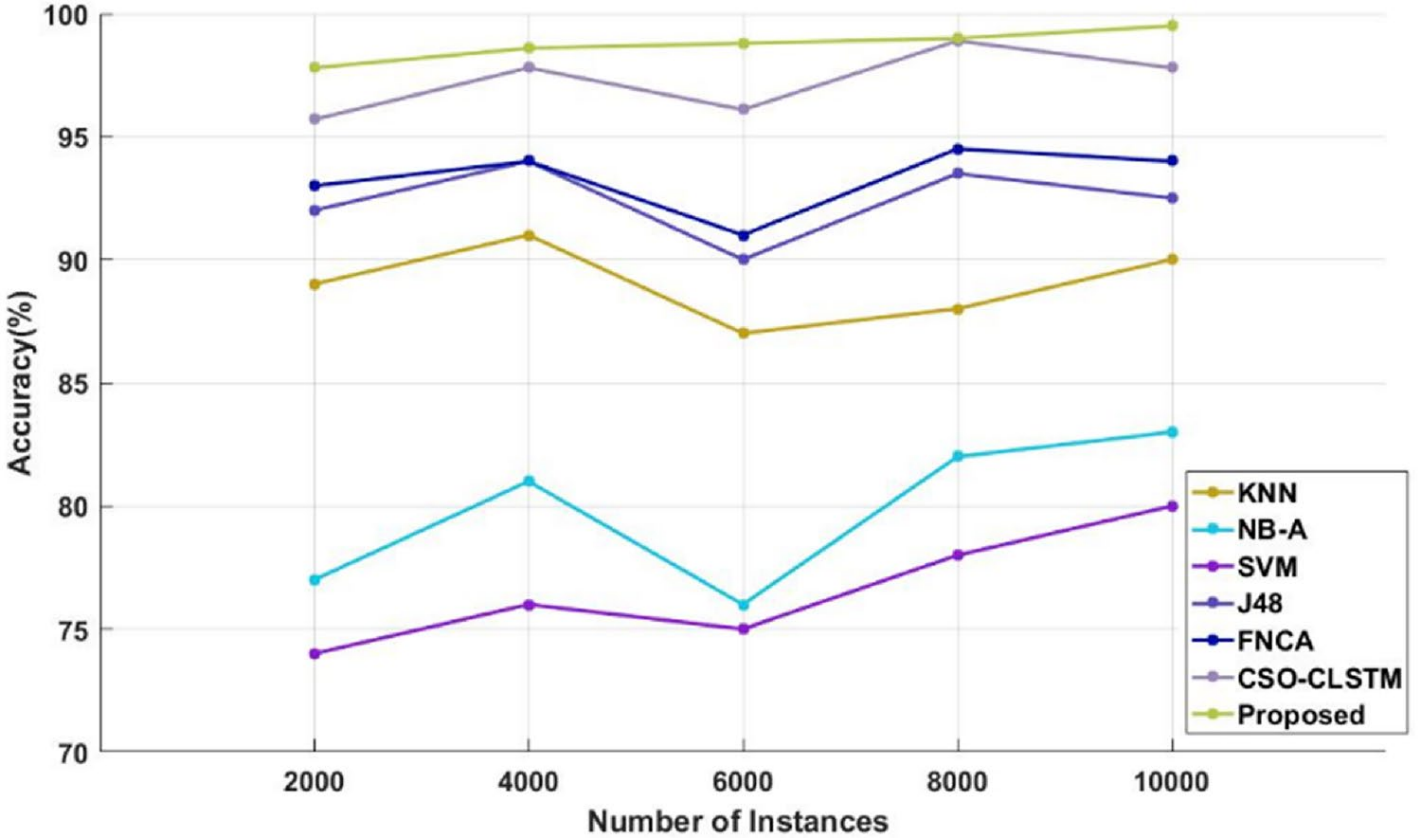
Accuracy analysis using diabetes disease dataset.

**Figure 11:**
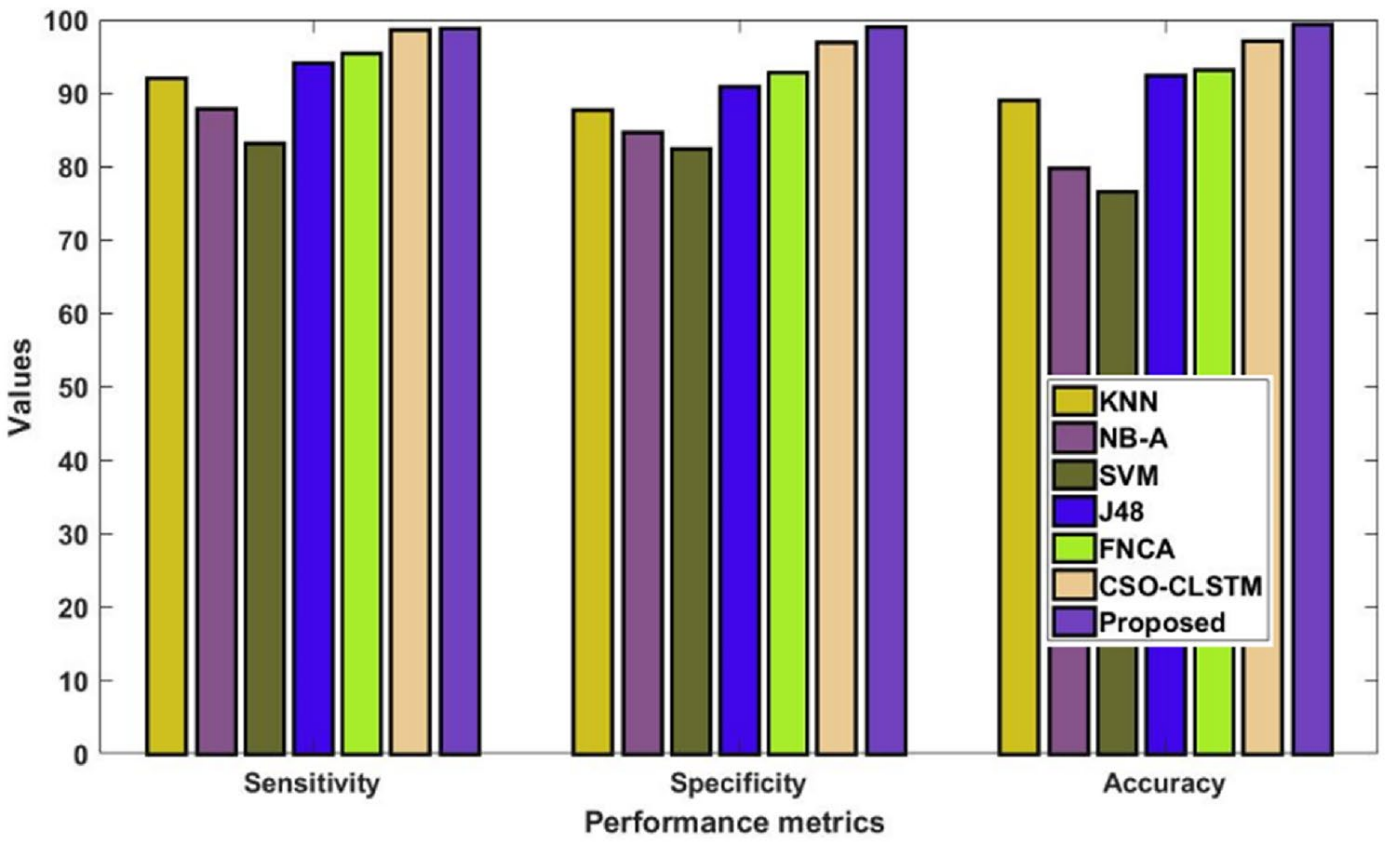
Overall comparative analysis using diabetes disease dataset.

Similarly, the efficacy of the existing and new categorization techniques is also verified and assessed using the Kaggle diabetes dataset. Tables 8, 9 and 10 present a comparison of the sensitivity, specificity, and accuracy of the traditional and proposed models using the Kaggle diabetes dataset. Figures 8, 9 and 10 then display the appropriate graphical results. These forecasts also demonstrate that the suggested DACL model performs better than earlier classification techniques with high performance values. Additionally, the overall performance evaluation makes use of the same diabetes data that is displayed in Table 11 and Fig. 11. With the inclusion of DAEM-based imputation and GFS optimization processes, the overall sickness diagnosis efficiency of the proposed model is much improved. Due to the fact that it facilitates the collection of data with balanced features required for precise illness classification. Additionally, the suggested DACL model greatly lengthens the classifier's training and testing time by restricting the subset of characteristics used for classification.

**Table 8:** Comparison based on sensitivity using diabetes disease dataset.

No of instances	KNN	NB-A	SVM	J48	FNCA	CSO-LSTM	Proposed
2000	92	87.50	83	93	94.50	98.10	98.5
4000	88	86	82.50	92	93.50	97.50	98.8
6000	92.80	88	83.80	93	94.50	98.90	99
8000	93.50	88	83	97	98	99.40	99.5
10,000	94.20	90	83.40	96	97	99.20	99.7

**Table 9:** Comparison based on specificity using diabetes disease dataset.

No of instances	KNN	NB-A	SVM	J48	FNCA	CSO-LSTM	Proposed
2000	84	83	82	92.50	94	98.80	99
4000	90	83	83	91	94.20	97.50	99
6000	87	86	83	93	94.10	96.90	99.2
8000	87.50	85	80	88	90.50	92	99.28
10,000	90	87	84	90.50	92	97.30	99.4

**Table 10:** Comparison based on accuracy using diabetes disease dataset.

No of instances	KNN	NB-A	SVM	J48	FNCA	CSO-LSTM	Proposed
2000	89	77	74	92	93	95.70	97.8
4000	91	81	76	94	94	97.80	98.6
6000	87	76	75	90	91	96.10	98.8
8000	88	82	78	93.50	94.50	98.90	99
10,000	90	83	80	92.50	94	97.80	99.5

**Table 11:** Performance comparative analysis using diabetes disease dataset.

Measures	KNN	NB-A	SVM	J48	FNCA	CSO-LSTM	Proposed
Sensitivity	92.10	87.90	83.14	94.20	95.50	98.62	98.8
Specificity	87.70	84.80	82.40	91	92.86	96.94	99
Accuracy	89	79.80	76.60	92.40	93.30	97.26	99.4

Sensitivity analysis of the data shows that the SVM model performed inefficiently in comparison to other traditional techniques. Furthermore, a significant degree of sensitivity was sought after above SVM by both the NB-A and KNN models. In the interim, the sensitivity produced by the J48 and FNCA techniques was comparable and competitive. When compared to SVM, KNN and NB-A models, the CSO-CLSTM model that was presented demonstrated better performance and yielded a high sensitivity value. Nonetheless, the proposed model performs better than every other strategy with satisfactory sensitivity. It is clear from examining the data in terms of specificity that the SVM model performed poorly in comparison to other current techniques. Furthermore, the KNN and NB-A models also made an effort to achieve better specificity than SVM. Moreover, the J48 and FNCA models produced competitive and near specificity. Furthermore, the CSO-CLSTM model acquired considerable specificity and high classification performance. However, the proposed approach acquired a high specificity of 99% in the presence of 2000 instances, while other models, including those derived using KNN, NB-A, CSO-CLSTM, SVM, J48, and FNCA techniques, only managed to achieve minimum specificity values as represented in Fig. 9 and Table 9. Also, the accuracy is estimated and compared with the aforementioned methodologies as demonstrated in Fig. 10 and Table 10.

**Figure 12:**
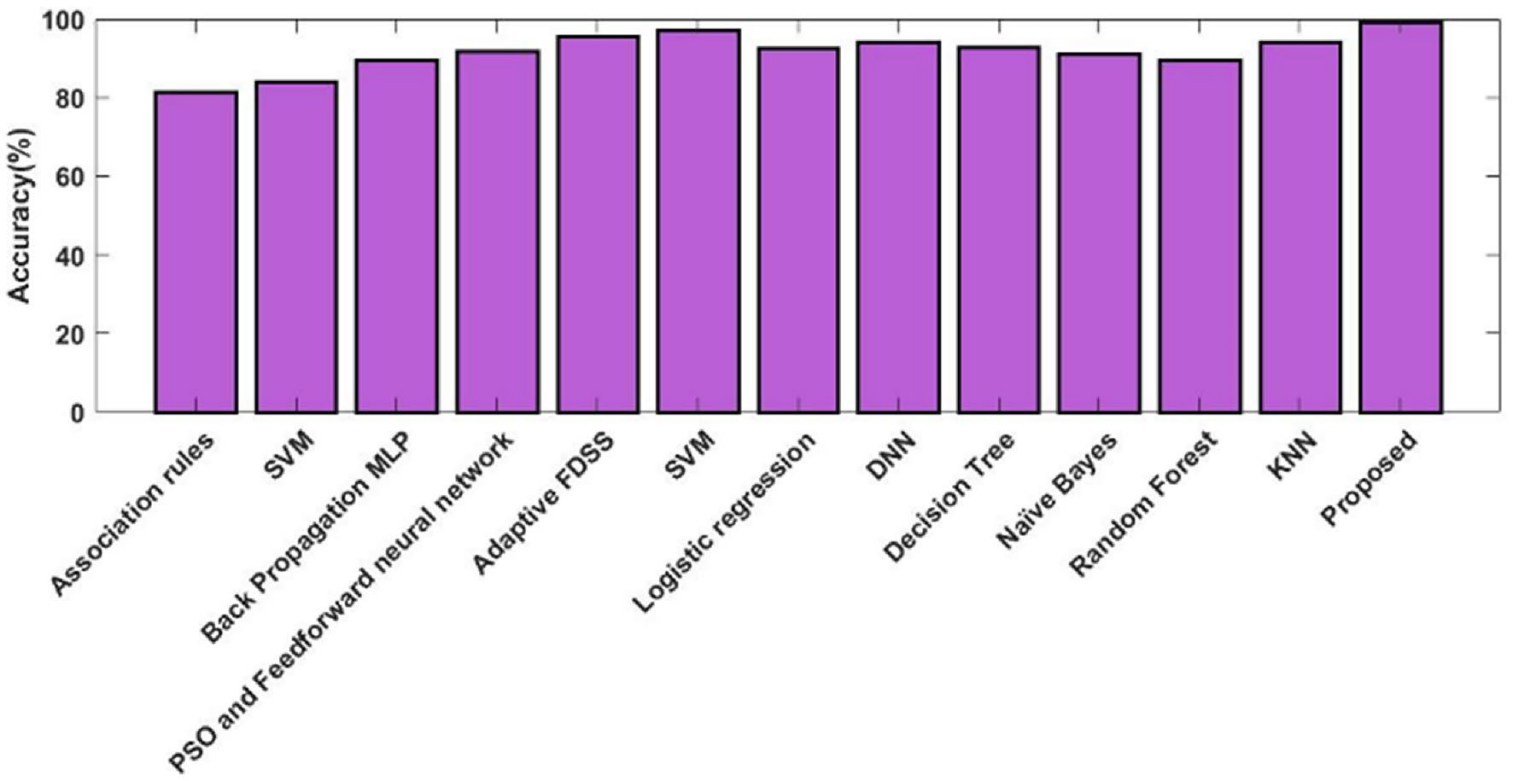
Training accuracy using diabetes dataset.

**Figure 13:**
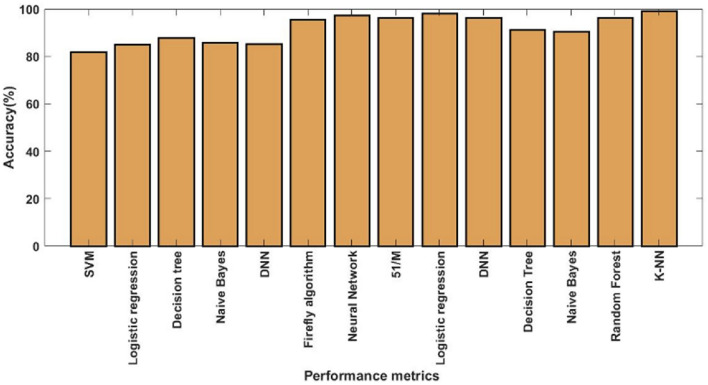
Testing accuracy using diabetes dataset.

Figures 12 and 13 show the training and testing accuracy values of hybrid models, recommended DOCL methodologies, and conventional machine learning methods using the diabetes dataset. Tables 12 and 13 then display its tabular values. The classifier's performance is validated and assessed using the training and testing accuracy scores. Results indicate that the proposed DACL model outperforms the baseline techniques, with testing accuracy rising to 99.2% and training accuracy to 99.4%.

**Table 12:** Comparison based on training accuracy.

Methods	Accuracy (%)
Association rules	81.51
SVM	84.12
BP-MLP	89.56
PSO-FFNN	91.94
Adaptive FDSS	95.56
SVM	97.36
LR	92.41
DNN	94.39
DT	92.76
NB	91.18
RF	89.41
KNN	94.28
Proposed	99.4

**Table 13:** Comparison based on testing accuracy.

Methods	Accuracy (%)
SVM	82
LR	85
DT	88.03
NB	85.86
DNN	85.20
FA	87.20
NN	95.55
SVM	97.41
LR	96.29
DNN	98.15
DT	96.42
NB	91.38
RF	90.46
KNN	96.42
Proposed	99.2

**Figure 14:**
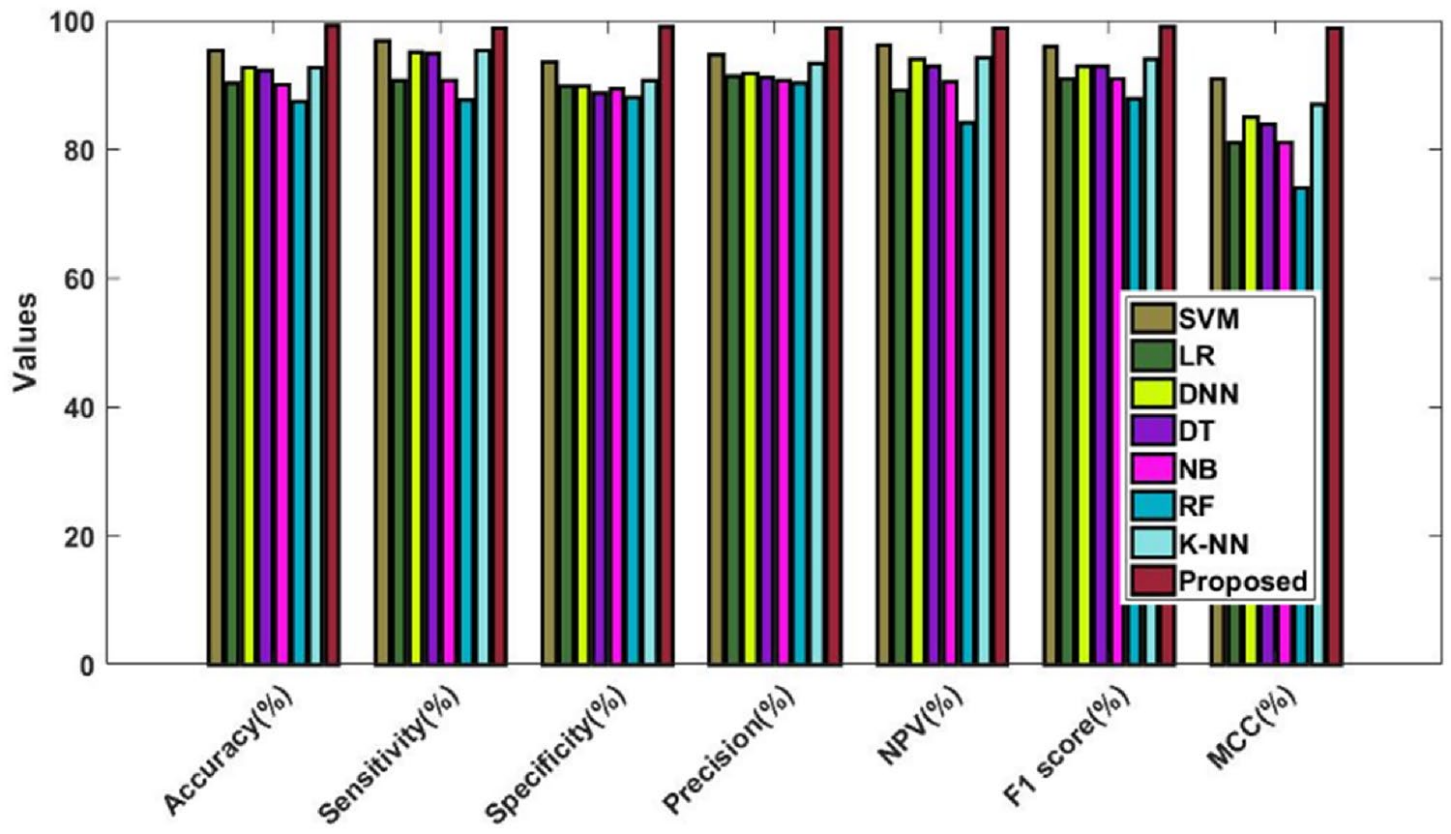
Overall comparative analysis using kaggle heart disease dataset.

**Figure 15:**
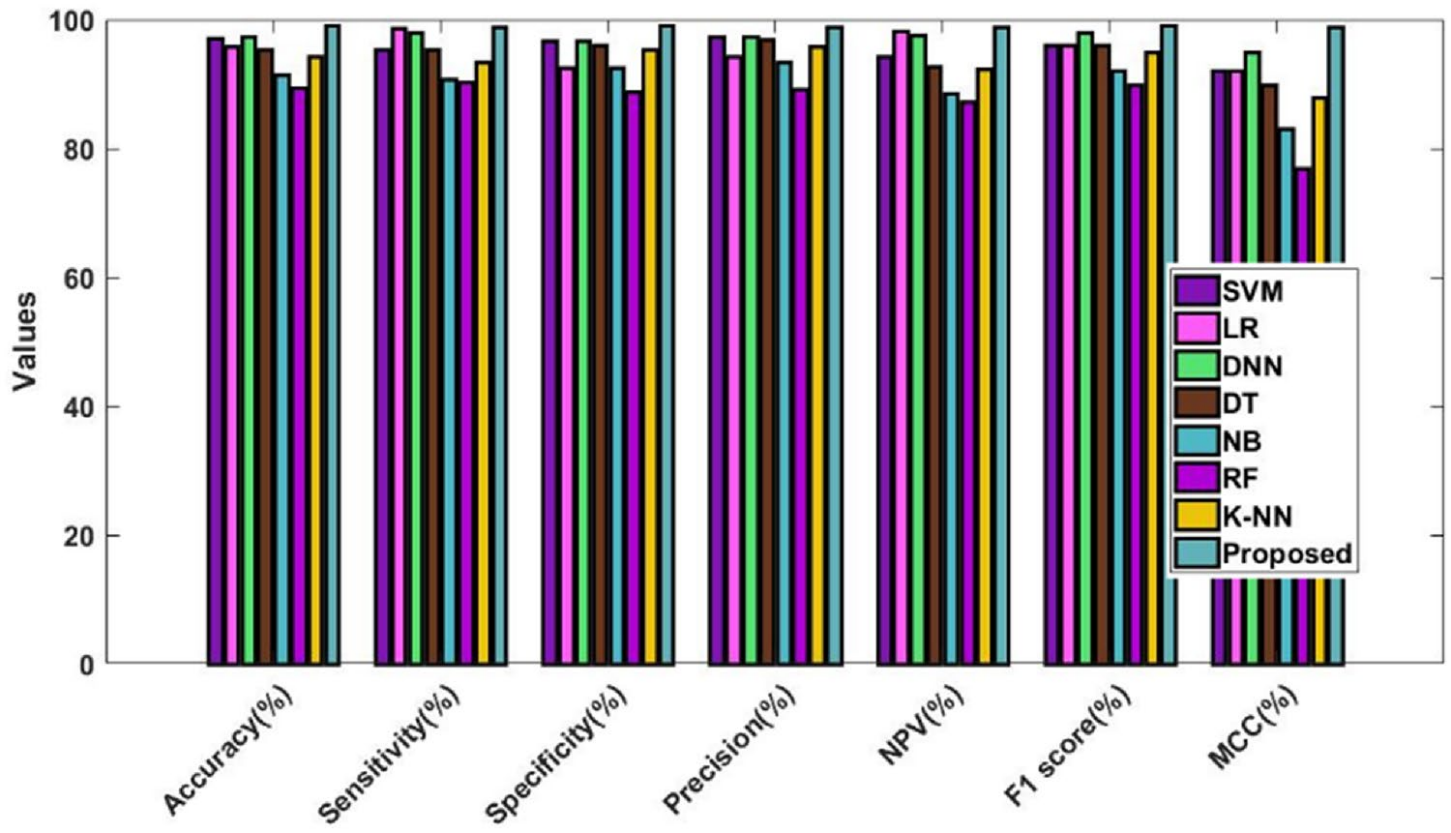
Overall comparative analysis using Statlog heart disease dataset.

Figure 14 and Table 14 validate and compare the overall outcomes of the suggested and conventional classification algorithms using the Kaggle heart disease dataset. As a result, the performance is validated and assessed using the Statlog dataset, as illustrated in Fig. 15 and Table 15. This study examines the performance outcomes and diagnosing efficacy of the proposed DACL using multiple types of datasets. Overall, the results demonstrate that all datasets can be handled by the recommended DACL with improved performance results. Given that the main factors influencing greater performance yields are DAEM, GFS, and ColBGaN inclusion.

**Table 14:** Comparison among the classification models using kaggle heart disease dataset.

Measures (%)	SVM	LR	DNN	DT	NB	RF	KNN	Proposed
Accuracy	97.04	95.93	97.41	95.37	91.38	89.48	94.25	99.2
Sensitivity	95.33	98.67	98	95.45	90.86	90.39	93.3	99
Specificity	96.67	92.50	96.67	96.11	92.42	88.78	95.46	99.1
Precision	97.28	94.27	97.35	96.85	93.39	89.33	95.89	99
NPV	94.31	98.23	97.48	92.79	88.56	87.18	92.34	99
F1-score	96	96	98	96	92	90	95	99.1
MCC	92	92	95	90	83	77	88	98.8

**Table 15:** Comparison among the classification models using Statlog heart disease dataset.

Measures (%)	SVM	LR	DNN	DT	NB	RF	KNN	Proposed
Accuracy	97.04	95.93	97.41	95.37	91.38	89.48	94.25	99.2
Sensitivity	95.33	98.67	98	95.45	90.86	90.39	93.31	99
Specificity	96.67	92.50	96.67	96.11	92.42	88.78	95.46	99.1
Precision	97.28	94.27	97.35	96.85	93.39	89.33	95.89	99
NPV	94.31	98.23	97.48	92.79	88.56	87.18	92.34	99
F1-score	96	96	98	96	92	90	95	99.1
MCC	92	92	95	90	83	77	88	98.8

**Figure 16:**
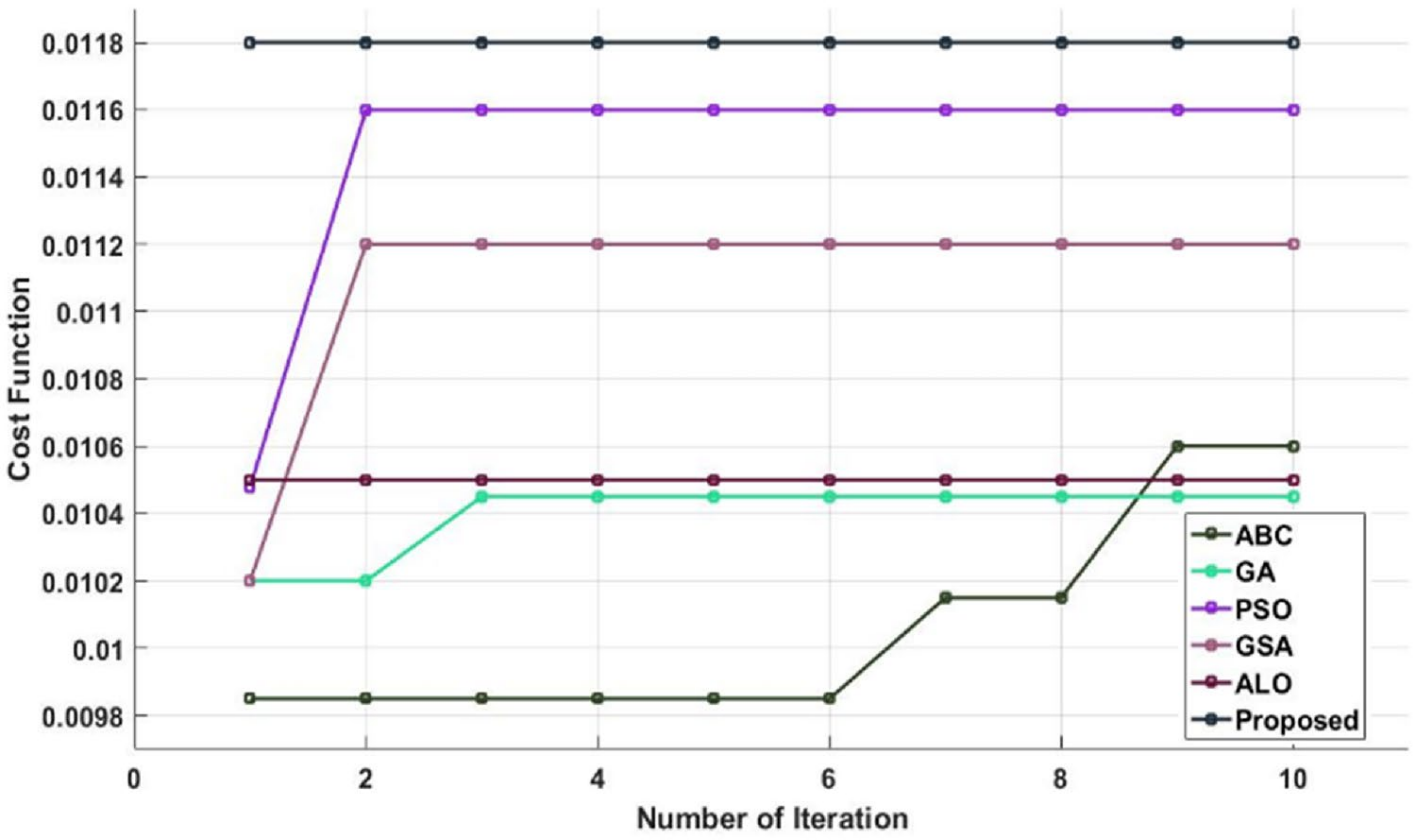
Cost function of different optimization techniques.

**Figure 17:**
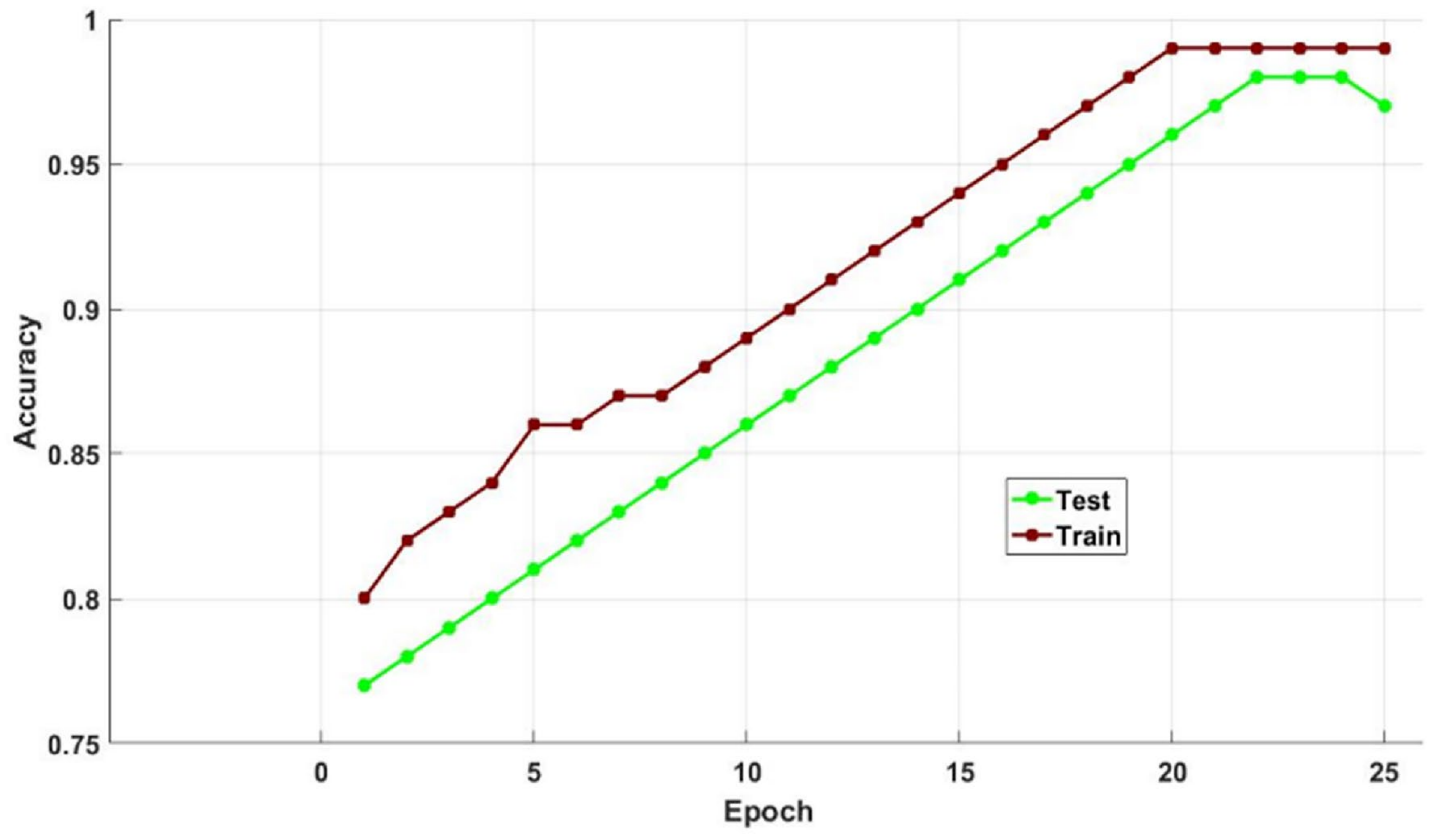
Training and testing accuracy with respect to varying Epochs.

**Figure 18:**
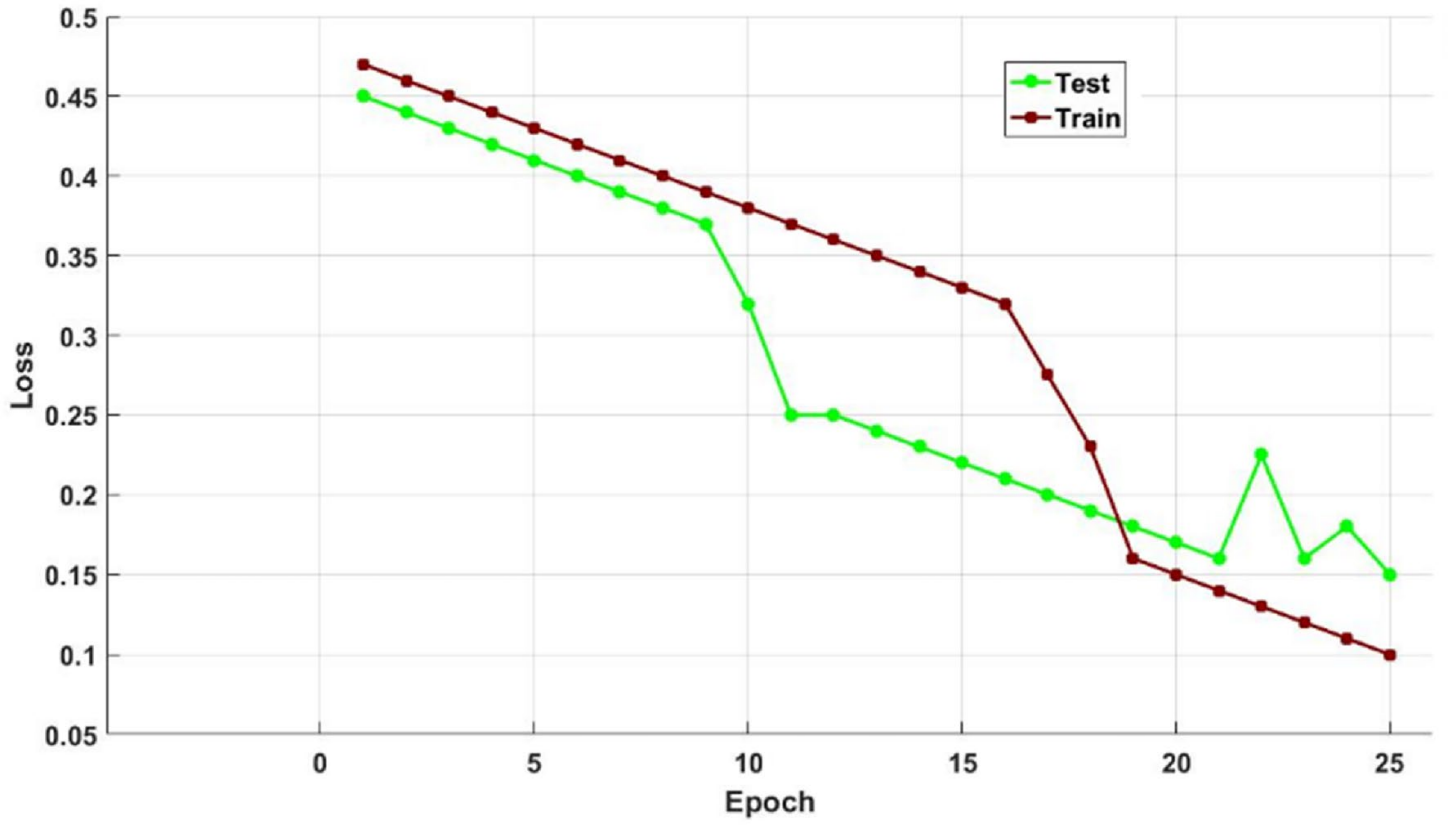
Training and testing loss values with respect to varying Epochs.

Figure 16 validates the cost function of existing and suggested optimization methods for different amounts of iterations. In order to determine the optimization performance of the suggested GSA technique, the cost function is verified and contrasted with the existing models in this study. According to the anticipated outcomes, the recommended GSA strategy performs infinitely better in terms of both cost and function than the current methods. Figures 17 and 18 also confirm and compare the training and testing accuracy and loss values of the proposed DACL technique with respect to varying epoch counts. The estimated results demonstrate that the suggested model gives higher accuracy with lower loss value by appropriately diagnosing the condition from the given data.

**Figure 19:**
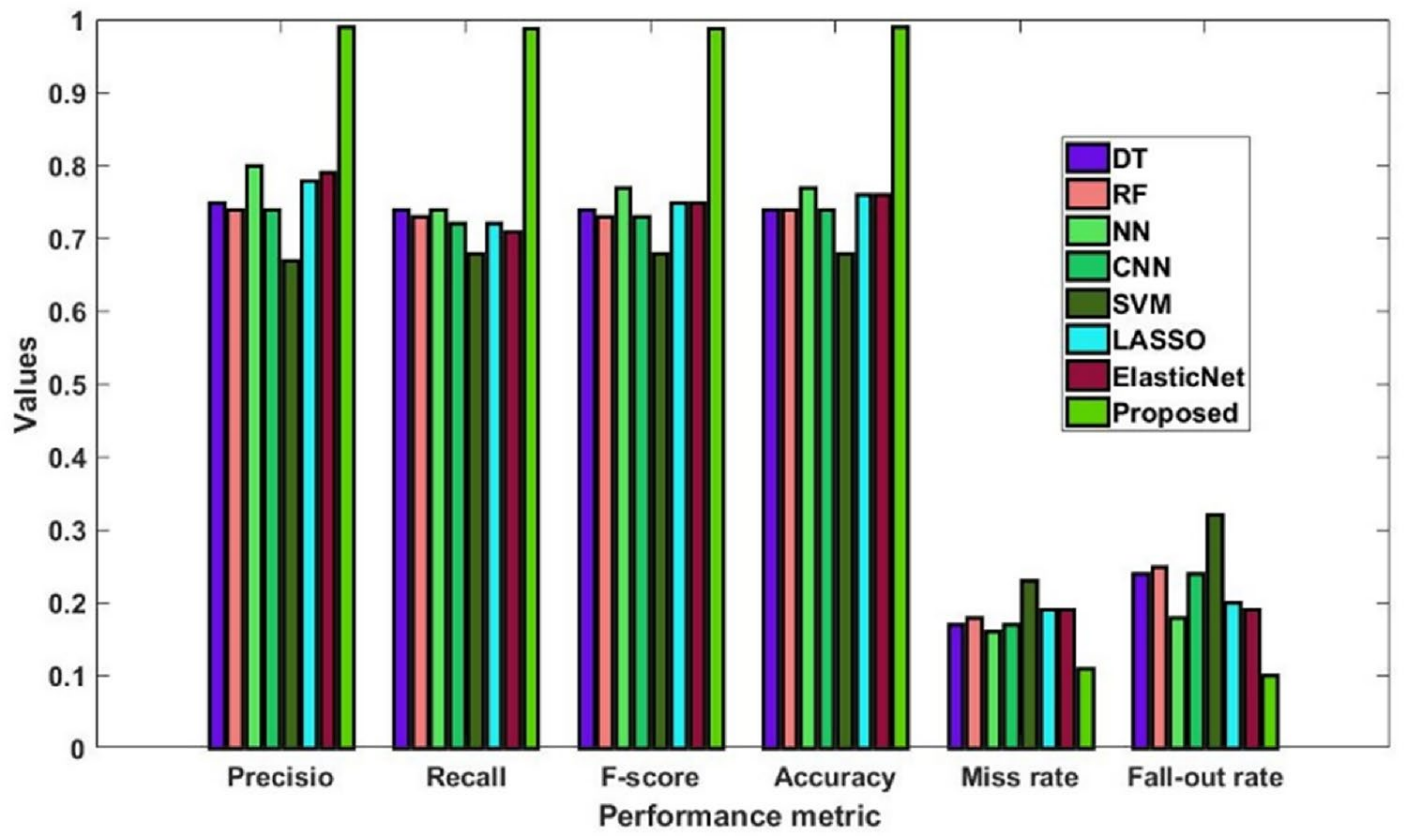
Overall performance analysis using stroke dataset.

**Figure 20:**
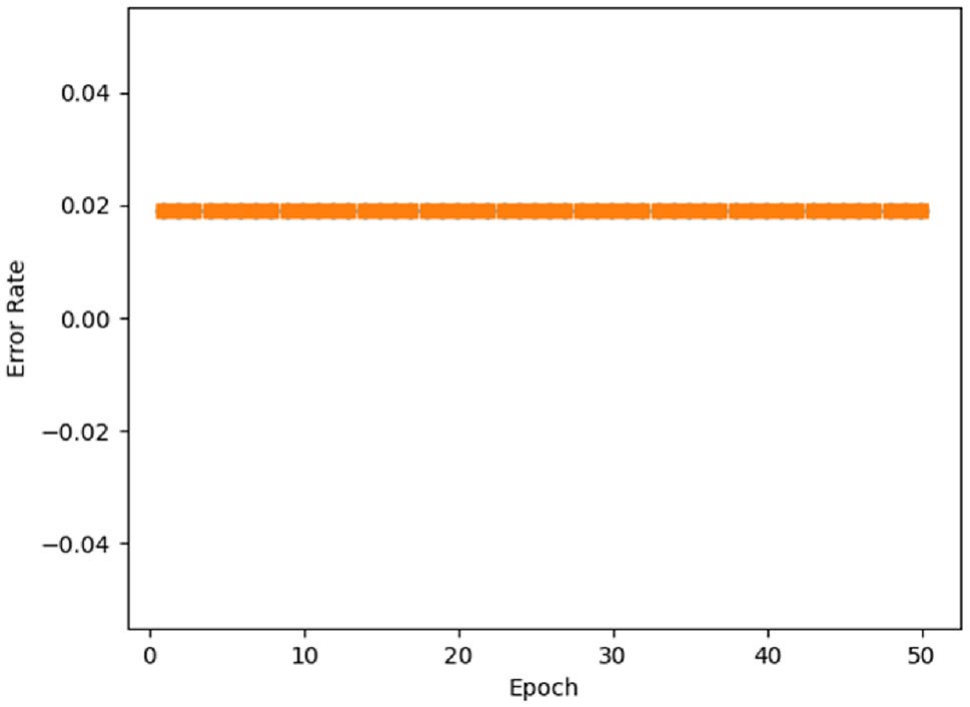
Error rate.

The overall comparative study of the traditional^49^ and novel methodologies utilizing the stroke dataset is presented in Fig. 19 and Table 16. To evaluate the efficacy of the proposed system, many performance measures are incorporated in this study. Figure 20 illustrates the classifier's error rate in relation to a range of epoch counts. Based on the analysis, it is concluded that the suggested categorization technique effectively lowers the mistake rate^50^.

**Table 16:** Comparative analysis using stroke dataset.

Methods	Precision	Recall	F1-score	Accuracy	Miss rate	Fall out rate
DT	75	74	74	74	17	24
RF	74	73	73	74	18	25
NN	80	74	77	77	16	18
CNN	74	72	73	74	17	24
SVM	67	68	68	68	23	32
LASSO	78	72	75	76	19	20
Elastic Net	79	71	75	76	19	19
Proposed	99	98.9	98.8	99	11	10

## Conclusion and future work

Developing an automated and enhanced AI-IoMT system for the diagnosis of different chronic diseases is the main objective of this project. The primary contribution of this study is the creation of an effective automated detection method that, using the medical data provided, can accurately identify and classify diabetes, heart disease, and stroke. In this work, a meta-heuristic model-based complex artificial intelligence system has been constructed for this aim. The three-tiered AI-IoMT architecture of the suggested framework first gathers the patient's medical data through sensors. The disease diagnosis is then carried out by the AI layer using cunning methods. After data collection, DAEM is utilized to complete the data cleaning and imputation procedures. An efficient deep learning architecture aids in locating the missing values for proper data processing and normalization. The GFO technique is used to identify the most significant characteristics from the imputed data in order to effectively diagnose diseases and classify their types. Processing can also be accelerated by reducing the dimensionality of the data. Furthermore, the newest ColBGaN classification algorithm is utilised to categorise the type of sickness based on specific features derived from patient data. Reduced computational complexity, flexibility to low-cost healthcare sectors, and accuracy in disease diagnosis are the primary benefits of the proposed DACL model. The effectiveness and development potential of the proposed DACL model were evaluated using several popular and publicly available datasets, including the Statlog, Kaggle, heart disease, and stroke datasets. The suggested framework was statistically evaluated in this study using well-known and pertinent performance evaluation standards, including F1-score, accuracy, precision, recall, sensitivity, specificity, and fall-out rate. The outcomes demonstrate that the proposed DACL performs better than the conventional approaches, with f1-score reaching 99%, precision and recall reaching 98.8%, and high accuracy reaching 99%. In the future, IoMT-supported biomedical systems may employ a set of deep learning algorithms for usage in hospital environments. To further shield IoMT-healthcare systems from modern cyberattacks, security might be bolstered.

Transfer learning models and other aspects of behavioral healthcare may be included in subsequent studies to put forward a behavioral health paradigm. Also, an intelligent model evaluation approaches could be introduced to evaluate the learnt models and improve the training methods' self-optimization. Security, anonymity, risk evaluation, standardization, seamless integration, and ethical issues in IoMT structures can be given top priority in future research. These elements are essential for encouraging the appropriate and efficient use of IoT technologies while guaranteeing the availability, privacy, reliability, and originality of medical data and systems. To combat hazards to the privacy of patient medical information, digital health applications require intelligent systems and frameworks. The future can be developed with a new method for cloud data interpretation leveraging the 5G network in the frame of a model for recommendation by utilizing deep learning and IoMT. Moreover, convoluted models with variational encoder can be used for the 5G cloud network application for healthcare data monitoring and analysis.

## Data Availability

The datasets used and/or analysed during the current study are available from the corresponding author on request.
